# Application of Ligature-Induced Periodontitis in Mice to Explore the Molecular Mechanism of Periodontal Disease

**DOI:** 10.3390/ijms22168900

**Published:** 2021-08-18

**Authors:** Peiya Lin, Hiromi Niimi, Yujin Ohsugi, Yosuke Tsuchiya, Tsuyoshi Shimohira, Keiji Komatsu, Anhao Liu, Takahiko Shiba, Akira Aoki, Takanori Iwata, Sayaka Katagiri

**Affiliations:** 1 Department of Periodontology, Graduate School of Medical and Dental Sciences, Tokyo Medical and Dental University (TMDU), Tokyo 113-8549, Japan; peiya.peri@tmd.ac.jp (P.L.); tsuchiya.peri@tmd.ac.jp (Y.T.); shimohira.peri@tmd.ac.jp (T.S.); liuperi@tmd.ac.jp (A.L.); shiba.peri@tmd.ac.jp (T.S.); aoperi@tmd.ac.jp (A.A.); iwata.peri@tmd.ac.jp (T.I.); katagiri.peri@tmd.ac.jp (S.K.); 2 Department of Lifetime Oral Health Care Sciences, Graduate School of Medical and Dental Sciences, Tokyo Medical and Dental University (TMDU), Tokyo 113-8549, Japan; komatsu.peri@tmd.ac.jp

**Keywords:** ligature, model mouse, periodontitis, systemic disease, immunity, microbiology, periodontal tissue, treatment

## Abstract

Periodontitis is an inflammatory disease characterized by the destruction of the periodontium. In the last decade, a new murine model of periodontitis has been widely used to simulate alveolar bone resorption and periodontal soft tissue destruction by ligation. Typically, 3-0 to 9-0 silks are selected for ligation around the molars in mice, and significant bone loss and inflammatory infiltration are observed within a week. The ligature-maintained period can vary according to specific aims. We reviewed the findings on the interaction of systemic diseases with periodontitis, periodontal tissue destruction, the immunological and bacteriological responses, and new treatments. In these studies, the activation of osteoclasts, upregulation of pro-inflammatory factors, and excessive immune response have been considered as major factors in periodontal disruption. Multiple genes identified in periodontal tissues partly reflect the complexity of the pathogenesis of periodontitis. The effects of novel treatment methods on periodontitis have also been evaluated in a ligature-induced periodontitis model in mice. This model cannot completely represent all aspects of periodontitis in humans but is considered an effective method for the exploration of its mechanisms. Through this review, we aimed to provide evidence and enlightenment for future studies planning to use this model.

## 1. Introduction

Periodontitis is an inflammatory disease caused by bacterial biofilms in the periodontal tissues [1]. Periodontitis generally occurs in adults, but it may also occur in children and adolescents [2]. According to research, 10–15% of the global population may be suffering from severe periodontitis. Severe periodontitis leads to tooth loss and has negative effects on other systemic diseases [3]. Therefore, it is important to explore the etiology of periodontitis and its treatment. In the past few decades, a variety of periodontitis models in mice have been established and effectively applied to the exploration of the mechanism of periodontitis and the effectiveness of new treatments [4,5]. Unlike other periodontitis models in mice, the ligature-induced periodontitis model shows acute alveolar bone loss and soft tissue inflammation in the initial period (within seven days), and the healing process of periodontitis can also be studied by ligature removal [6,7]. Because of the technical difficulty of placing the ligature around the mouse teeth, this model was not widely used until the image and detailed description of the method were first published in 2013 by Abe et al. [8]. Furthermore, additional bacterial administration in mice with ligature-induced periodontitis showed an increase in the severity of periodontitis [9,10].

The purpose of this review is to summarize the findings in the ligature-induced periodontitis model in mice: the interaction between systemic diseases and periodontitis, biological evidence in periodontal tissue, systemic effects, immunological and bacteriological mechanisms, and new treatments for periodontal disease. We are trying to provide relevant information for future studies planning to use the ligature-induced periodontitis model in mice to investigate the molecular mechanisms of periodontal disease.

## 2. Ligature-Induced Periodontitis in Mice

During induction of experimental periodontitis in mice by ligation, plaque is accumulated around the ligature, and the placement of the ligature causes a continuous inflammatory infiltration, which leads to the destruction of periodontal tissue within seven days. According to the protocol from Abe et al., we summarized the equipment and procedures of ligation for reference. For the novel procedure, the following tools are needed: Dumont forceps (Fine Science Tools, North Vancouver, BC, Canada), Dumont cover slip forceps (Fine Science Tools), Spring scissors (Fine Science Tools), and 5-0 silk suture (Roboz Surgical Instrument Co., MD, USA). To perform ligation, Dumont forceps are used to pass 5-0 silk suture through the gap between the second and third molars, as well as the gap between the first and second molars. Then, looping around the second molar with forceps occurs. In the end, tying the suture firmly by a triple-knot and cutting the excess suture by Spring scissors occurs [8]. In recent years, some researchers have used nylon thread or steel wire for ligation [6,11,12].

In fact, oral administration of bacteria and local injection of lipopolysaccharide (LPS) or *Porphyromonas gingivalis* are also commonly used methods for establishing periodontitis in mice [13]. However, these two methods take a longer time (around four to six weeks) to produce a significant loss of periodontal tissue than the ligation method [14,15]. Therefore, ligature-induced experimental periodontitis is suitable for studying the destruction of periodontal tissue. The destruction of periodontal tissue in human periodontal disease is originated by microorganisms. The host immune response against bacteria is considered to play a very important role in the progression of periodontal tissue destruction [16]. The ligature-induced periodontitis model in mice is beneficial to observe the host response, because mice display fewer individual differences than humans [4,17].

## 3. Evaluation in Periodontal Tissues

### 3.1. Evaluation in Gingiva

Periodontal disease is a prevalent disease that results in the loss of gingival tissue, which provides the first line of defense against various environmental and microbial irritants. In particular, epithelial cells are interconnected by tight junctions, adherence junctions, desmosomes, and gap junctions, forming an epithelial barrier at the surface layer of the gingival tissue [18,19,20]. Recent studies have indicated the unique roles of Grainyhead-like (Grhl) family genes in the regulation of epithelial barrier functions [21,22,23,24,25,26], and periodontitis was induced in *Grhl2* conditional knockout (cKO) mice using a 5-0 silk ligature. *Grhl2* cKO suppressed the expression of junction proteins at the junctional epithelium and promoted the progression of alveolar bone loss in the ligature-induced periodontitis model. Increased epithelial penetration of oral bacteria was observed in *Grhl2* KO mice compared to WT mice. In addition, a significant increase in the blood loadings of oral bacteria was observed in *Grhl2* KO mice compared to WT littermates [27]. Meanwhile, the destroyed epithelium was observed by hematoxylin and eosin staining of the gingiva in ligated WT mice. mRNA expression of the secreted glycoprotein, collagen triple helix repeat containing 1 (CTHRC1), was also shown to be highly expressed in the gingiva. Increasing evidence has indicated its suppressive effect on inflammation [28,29,30], suggesting that CTHRC1 may play an important role in the regulation of periodontal inflammation [31].

In addition to the above, ligature model mice have been observed to induce inflammation in the gingiva, using a novel intravital endoscopic technology [32]. In periodontal inflammation, the gastrin-releasing peptide (GRP), a neuropeptide with growth-stimulatory and tumorigenic properties, has been reported to be an important factor in the complex cascade of chemical activity [33,34]. GRP expression in the gingival tissue of mice subjected to ligature was examined histologically, and GRP-positive cells were mostly observed in the oral epithelium [35]. Additionally, gingival inflammation has been identified as a key factor that initiates drug-induced gingival overgrowth (DIGO), which is a side effect of phenytoin, nifedipine, and cyclosporine A (CsA) [36,37]. To investigate the role of gingival inflammation in DIGO, the ligature model mice were administered CsA daily. After 28 days, a significant increase in the degree of gingival overgrowth and expansion of the connective tissue area was observed, whereas cessation of CsA reduced gingival overgrowth. Moreover, the administration of an antibiotic cocktail, which suppressed the expression of these inflammatory cytokines in the gingiva, attenuated gingival overgrowth induced by ligation and CsA. Interestingly, thin ligatures (7-0) induced weaker TNF-α, IL-1β, and IL-6 mRNA expression and less gingival overgrowth than thick ligatures (5-0) [38].

Comprehensive proteomic analysis of the gingival tissue of ligatured mice was performed using pressure cycling technology-assisted mass spectrometry. A total of 1614 proteins with ≥2 peptides were quantified, with an estimated protein false discovery rate of 0.06%. The gingival tissue protein abundance was shown to be mainly dependent on the progression of periodontitis by unsupervised clustering analysis. Additionally, over-representation of innate immune regulation, signal transduction, and homeostasis processes was revealed by gene ontology (GO) enrichment analysis [39]. Moreover, although ferritin, an iron-binding protein, was detected in the gingival epithelium and gingival connective tissue by immunochemical staining, the intensity of positive staining became significantly stronger along with the extent of inflammatory infiltration [40].

Additionally, during periodontal disease, reactive oxygen species (ROS) are produced by the host cells as a defensive response to bacterial pathogens [41]; however, extensive ROS production (oxidative stress) induces damage to DNA, proteins, and lipids in the host tissue [42]. The ligature model was used to visualize the oxidative stress induced by experimental periodontitis [43].

### 3.2. Evaluation in Bone

Bone homeostasis is maintained by a continuous physiological process termed bone remodeling. This process requires the balanced activity of osteoblasts and osteoclasts. Excessive osteoclast activity leads to pathological bone resorption, including periodontitis [44]. Since ligature-induced periodontal models cause severe alveolar bone destruction, they have been used in many studies to reveal the pathological mechanisms or to discover a treatment strategy for periodontitis.

#### 3.2.1. Osteoclasts

Most of these studies have focused on osteoclasts and have reported several genes or molecules related to osteoclastogenesis. Mature osteoclasts (OCs) are multinucleated giant cells. Many molecules have been implicated in cell fusion. OC-stimulatory transmembrane protein (OC-STAMP), dendritic cell-specific transmembrane protein (DC-STAMP), and permissive fusogen CD9 are involved in osteoclast fusion [45]. Although *Dcstamp* KO mice showed an osteopetrotic phenotype, *Ocstamp* KO mice showed no difference in systemic bone density. However, ligature-induced bone resorption was impaired in *Ocstamp* KO mice compared to WT mice. Systemic administration of anti-OC-STAMP-mAb suppressed the expression of CD9 mRNA, but not of DC-STAMP mRNA [46]. These findings have demonstrated that OC-STAMP partners CD9 to promote periodontal destruction by upregulation of fusion during osteoclastogenesis, suggesting that anti-OC-STAMP-mAb may lead to the development of a therapeutic regimen for pathological bone destruction such as periodontitis.

High mobility group box 1 protein (HMGB1) is a non-histone DNA-binding protein that is secreted in various inflammatory diseases such as periodontitis [47]. Administration of an anti-HMGB1 neutralizing antibody in an experimental periodontitis model attenuated alveolar bone resorption and inflammatory cytokines [48].

Phosphatase and tensin homolog (PTEN), a specific tumor suppressor gene, limits PI3K activity and reduces downstream serine/threonine kinase AKT signaling [49]. Expression of PTEN mRNA was reduced and inflammatory cytokines were increased in ligature-induced periodontitis mice. However, overexpression of PTEN in experimental periodontitis mice attenuated the expression of IL-1, IL-6, and TNF-α. PTEN inhibits inflammatory bone loss in periodontitis [50].

Neurotrophin receptor-interacting MAGE homologue (NRAGE)-deficient mice with ligature-induced periodontitis exhibited severe alveolar bone loss compared to control mice. Knockout of NRAGE induced autophagy-related gene expression and accelerated bone destruction by increasing the activity and differentiation of osteoclasts [51].

The triggering receptor expressed on myeloid cells-1 (TREM-1) is known to modulate local and systemic inflammation [52]. TREM-1 expression in the gingiva was upregulated at the ligated sites in a time-dependent manner. Local administration of the TREM-1 inhibitor LP17 significantly suppressed alveolar bone loss. Furthermore, LP17 significantly downregulated the IL-17A and RANKL/OPG ratios. TREM-1 regulates the IL-17A-RANKL/OPG axis and bone loss in experimental periodontitis [53].

The SLIT2 protein, a member of neuronal guidance cues, has been reported to regulate inflammation and cancer progression [54]. SLIT2 expression was upregulated in periodontitis in both humans and mice, and a higher expression of SLIT2 accelerated the progression of periodontitis. According to the RNA sequencing data of *Slit2*-Tg-mice, *Slit2* overexpression upregulated the expression of *Robo1* and *Robo2*, which encodes the receptor of the SLIT2 ligand. Furthermore, GO pathway enrichment analysis and western blot analysis showed the activation of the MAPK signaling pathway [55].

Adaptor protein SH3-domain binding protein 2 (SH3BP2) plays a critical role in the inflammatory response and osteoclastogenesis of myeloid lineage cells through spleen tyrosine kinase (SYK) [56]. Ligature-induced alveolar bone loss in *Sh3bp2*^−/−^ mice was lower than that in *Sh3bp2*^+/+^ mice. Furthermore, myeloid cell-specific SYK deletion decreased ligature-induced periodontitis without affecting both inflammatory cytokine expression and osteoclast induction. Administration of the SYK inhibitor GS-9973 restored ligature-induced alveolar bone loss by suppressing osteoclast differentiation and function. These data suggested that the SH3BP2/SYK signaling axis regulates bone loss in periodontitis and that SYK can be a potential therapeutic target for alveolar bone loss in periodontitis [57].

The aryl hydrocarbon receptor (AhR)-ligand axis is involved in inflammatory responses and bone homeostasis [58]. In this study, the expression of cytochrome P450 subfamily B member 1 (CYP1B1), the AhR target gene cytochrome, and the roles of 6-formylindolo[3,2-b]carbazole (FICZ) were investigated. The expression of CYP1B1 was reduced in periodontitis. Both systemic and topical application of FICZ improved ligature-induced bone loss. Furthermore, FICZ treatment ameliorated the expression of pro-inflammatory cytokines. In vitro experiments revealed that FICZ pre-treatment reduced LPS-induced inflammation in periodontal ligament cells via increased phosphorylation of STAT3. FICZ also promoted the mineralization of periodontal ligament cells (PDLCs) via activation of the Wnt/β-catenin signaling pathway [59].

The P2X family purinergic receptor P2X5 has been implicated in bone biology [60]. *P2rx5*-deficient mice had decreased ligature-induced bone loss compared to WT littermates. Gene expression analysis of gingival tissue of ligated mice showed that *Il1b*, *Il6*, *Il17a*, and *Tnfsf11* expression levels were significantly reduced in *P2rx5*-deficient mice. This study implied that the P2X5 receptor regulates bone loss relative to periodontitis and may be used as a novel therapeutic target [61].

Inflammasomes are multi-protein complexes assembled by intracytoplasmic pattern recognition receptors, such as toll-like receptors (TLRs). The NOD-like receptor family pyrin domain containing 3 (NLRP3) inflammasome is a typical inflammasome. Alveolar bone loss was reduced in Nlrp3KO mice compared to WT mice in the experimental periodontitis model. The *Lysm-Cre/Rosa*^nTnG^ mouse, a double-fluorescent Cre reporter mouse, was used to observe the effects of the NLRP3 specific inhibitor, MCC950, on *Lysm-Cre*^+^ osteoclast precursor cells in vivo. The MCC950-treated periodontitis group had a lower number of *Lysm-Cre*^+^ cells compared to the vehicle-treated periodontitis group. Therefore, NLRP3 deficiency and MCC950 reduced the number of osteoclast precursors and prevented osteoclastogenesis [62].

The transient receptor potential vanilloid 1 (TRPV1) channel belongs to the transient receptor potential superfamily [63]. *Trpv1*^−/−^ mice developed severe bone loss in an experimental model of periodontitis. TRPV1-mediated calcitonin gene-related peptide (CGRP) release in gingival tissues suppressed LPS-induced osteoclastogenesis [64].

MicroRNAs (miRNAs), small non-coding RNAs that are rarely translated into peptides or proteins, are focused on various diseases. One miRNA, miR-335-5p, has been reported to have osteogenic activity and inhibits bone-related disorders [65]. The transgenic mice (335-Tg) overexpressing miR-335-5p driven by the osterix promoter were subjected to a ligature-induced periodontal model (EP). Alveolar bone loss was reduced in 335-Tg-EP mice compared to WT-EP. Additionally, the qPCR analysis revealed that the expression levels of *Il1b*, *Il6*, *Tnfa*, and *Tnfsf11* were reduced in the alveolar bone and gingival tissue of 335-Tg-EP mice compared to WT-EP mice. These results indicated that MiR-335-5p expression negatively regulates periodontal inflammation, thereby preventing ligature-induced bone loss [66].

#### 3.2.2. Osteocytes

Osteocytes are terminally differentiated osteoblast lineage cells and are embedded within mineralized bone. Osteocytes act as a commander for bone metabolism by overseeing the functions of osteoblasts and osteoclasts. Osteocytes are known to be a major source of RANKL [67], an essential molecule in osteoclastogenesis, and sclerostin, a negative regulator of osteoblast activity and bone formation through the Wnt/β-catenin pathway [68].

Dickkopf-1 (Dkk-1) is an inhibitor of the Wnt signaling pathway that is expressed in various organs and several cell types. Osteocyte-specific *Dkk1*-deficient mice (*Dkk1*^fl/fl^; *Dmp1*^Cre^) significantly prevented ligature-induced bone loss and mitigated inflammation. The expression of Wnt target gene T cell factor (TCF)-7 and lymphoid enhancer factor (LEF)-1 was decreased in ligated sites compared to non-treatment sites in WT mice. On the other hand, in osteocyte-specific *Dkk1*-deficient mice, the expression of TCF-7 remained at the control level, and the expression of LEF-1 was not changed compared to WT mice. In this study, Dkk-1 derived from osteocytes played a crucial role in alveolar bone loss in periodontitis [69].

#### 3.2.3. Others

Several studies have implied that aging and sex affect ligature-induced periodontitis. Surveys in the United States suggest that male subjects are more prone to developing periodontitis [70]. However, there are no reports of sex as a risk factor for periodontitis. In this study, both periodontal murine models (oral gavage and ligature models) were used to determine whether sex was a risk factor for periodontitis. In contrast to the results in humans, both murine models showed that female mice developed periodontitis at a higher progression rate [71]. Mesenchymal stem cells (MSCs) play important roles in the repair of damaged tissues. However, aging is known to impair the function of MSCs. In a previous study, a ligature-induced periodontal mouse model was utilized to investigate the aging-induced impairment of MSC function. Micro-CT and histological analysis revealed more severe bone loss associated with increased osteoclast activity in aged (50-week-old) mice than in young (5-week-old) mice. Immunohistological analysis revealed that the number of MSCs (PDGFR-positive) was reduced at the periodontal sites in aged mice. An in vitro study revealed that the expression of surface antigen markers of MSCs (Sca-1, CD90, CD146), colony formation, migration, and osteogenesis of aged MSCs were significantly lower than those of young MSCs. These results suggested that aging-induced impairment of MSC function is potentially correlated with progressive periodontal tissue deterioration [72]. Recently, the ligature-induced bone loss method has been modified for dental implants to investigate peri-implantitis. Eight weeks after molar extraction, screw-shaped machined-surface titanium implants were placed in the extracted sites and allowed to osseointegrate for four weeks. After the confirmation of osseointegration, a 6-0 silk ligature was placed around the implant and kept for one or two weeks. Micro-CT analysis revealed ligature-induced bone loss in peri-implant sites, such as periodontitis. Furthermore, the ligature was removed after one week; the periodontitis group experienced significant bone gain, whereas the peri-implantitis group did not. These results implied that peri-implant tissue may recover more slowly than periodontal tissue [7,73]. This modified method may be useful for exploring the differences in periodontitis and peri-implantitis, thereby leading to the elucidation of the mechanisms of peri-implantitis.

### 3.3. Evaluation in Periodontal Ligament Cells

The periodontal ligament (PDL) is a connective tissue that connects the cementum and the alveolar bone, which supports the tooth by buffering the masticatory force and contributing to tooth nutrition. The collagen fibers of the PDL secure the tooth in the bone socket. When the degradation of PDL collagen fibers occurs due to chronic inflammation of periodontitis, alveolar bone decreases, resulting in eventual tooth loss. Picrosirius red (PSR) histological staining of PDL showed a significant reduction in total collagen content 5 days after ligation compared to control values, although they recovered after 14 days of ligation. Additionally, thin fibers were significantly increased in ligated mice at 5 and 14 days, suggesting the promotion of collagen fiber remodeling in the PDL of periodontitis mice. However, inhibition of transglutaminase (TG) activity that has been indicated to regulate collagen fiber formation [74] increased total collagen and thick collagen fiber content in the group with ligation at 5 days [75].

Long non-coding RNAs (lncRNAs) are a class of non-protein-coding transcribed RNAs. lncRNA H19 has been shown to be an important factor in autophagy [76,77] and has attracted attention for its interaction with periodontitis [78,79]. In PDLCs of ligated mice, autophagy was significantly increased and H19 expression was also significantly upregulated during inflammation [80].

### 3.4. Summary

The contents of this section are presented in Table 1. Periodontitis is a complex disease, as many pathogenic factors interact between the surrounding tissues, including both soft and hard tissues. Therefore, the pathological mechanisms underlying periodontitis have not been fully elucidated. Herein, ligature model mice were used to examine the effects of ligature-induced periodontitis on periodontal tissues; however, these studies mainly focused on individual tissues and cells. In addition, no study has investigated the effect of periodontitis on cementum, which is another important periodontal tissue, using a ligature model.

Regarding the efficacy of ligature model mice in observing the response of periodontal tissue against periodontitis, the model has become prevalent, and it seems to be sufficiently established.

## 4. Ligature-Induced Periodontitis in Disease Model Mice

There are many reports on the relationship between periodontal disease and systemic diseases [81]. However, the underlying mechanism is still unclear, and it is difficult to demonstrate the mechanisms by human epidemiological and/or intervention studies. In this section, we review the novel findings by the application of ligature-induced periodontitis in disease model mice.

### 4.1. Diabetes and Obesity Model Mice with Ligature-Induced Periodontitis

Many researchers are interested in the relationship among periodontal disease, diabetes, and obesity. Type 2 diabetes patients with poor glycemic control [82,83] and obese patients [84,85] showed a more severe and higher incidence of periodontitis. In accordance with previous epidemiological findings in humans, we previously reported that significantly severe alveolar bone loss (ABL) was observed in streptozotocin-induced diabetic (STZ) mice compared to wild-type (WT) mice at seven days post-ligation. Histological analysis showed lower alkaline phosphatase activity in STZ mice. In addition, an increased number of tartrate-resistant acid phosphatase-positive multinucleated cells were observed at the ligated sites in STZ mice. We suggested that an imbalance in bone metabolism caused osteoclastogenesis in insulin-deficient diabetes [86]. Another study also reported that mice fed a high-fat diet for 8 weeks with silk ligature ligation showed higher ABL compared to those fed a normal chow diet. Although ABL and periodontal osteoclast numbers were not affected by diet regardless of ligation or sham-ligation, leukocyte and macrophage numbers and protein levels of tumor necrosis factor α (TNF-α) in the periodontium and serum interleukin (IL)-6 levels were downregulated in periodontitis mice fed a high-fat diet. These findings indicated that an impaired immune response occurred both periodically and systemically in pre-obesity overweight mice [87]. They also evaluated the effects of obesity on macrophage infiltration and activation in periodontal tissue with periodontitis. A 16-week high-fat diet-induced obesity mouse model was constructed, and periodontitis was induced by ligation for 10 days. ABL increased significantly with periodontitis and obesity. F4/80 and monocyte chemotactic protein 1 (MCP1) expression was significantly upregulated in gingival tissues with periodontitis; however, it was significantly downregulated in the context of obesity. These results suggested that obesity may paralyze the innate immune response of the periodontium by attenuating the infiltration and activation of macrophages, and further aggravate periodontal disease [88]. They concluded that when analyzing the relationship between obesity and periodontitis, the confounding effects of an imbalanced postoperative weight loss (POWL) should be considered. Combined mouse models of diet-induced obesity using a high-fat diet and ligation-induced periodontitis were evaluated. Without considering POWL as a confounding factor, conflicting results, including contradictory changes in high-density lipoprotein (HDL) cholesterol caused by obesity or periodontitis, and unequal levels of fasting serum glucose, total cholesterol, and HDL cholesterol were observed in the sham-ligation controls [89]. Another study investigated the role of polymorphonuclear neutrophils (PMNs) in mediating diabetic tissue damage to the periodontium in a model of chronic hyperglycemia in Akita mice. In addition to severe bone loss in Akita mice, chronic hyperglycemia predisposes to an exaggerated inflammatory response and primes leukocytes for marginalization and superoxide production, but not for transmigration [90]. In addition, ligature-induced periodontitis was applied to C57BL/6-*db/db* and inbred C57BL/6 mice to investigate the effects of periodontitis on the function of pancreatic β-cells with proinflammatory cytokine-related immune mechanisms. Pancreatic β-cell failure, with insulin resistance, was observed in *db/db* mice, while periodontitis could aggravate β-cell dysfunction-related features [91]. Recently, metabolome analysis has been applied to ligature-induced periodontitis in mice with diet-induced obesity. Of the 2247 reference features, the presence of periodontitis altered 165 features in normal chow diet-treated lean mice, although 885 features were altered in mice fed a high-fat diet for 16 weeks. A high-fat diet altered 525 features in the absence of periodontitis, but 1435 in the presence of periodontitis. Compared with healthy counterparts, periodontitis and a high-fat diet had distinct effects on the gingival metabolome. The metabolomic impact of periodontitis was generally greater in high-fat diet mice than in lean controls [92]. Another study performed fecal microbiota transplantation and ligature-induced periodontitis in mice with high-fat diet-induced obesity and demonstrated that gut dysbiosis-associated metabolites from high-fat diet-fed mice worsen alveolar bone destruction. Fecal metabolomics revealed an elevated purine degradation pathway activity in mice fed a high-fat diet, and recipient mice had elevated levels of serum uric acid upon induction of periodontal disease. Furthermore, ligature-induced periodontitis caused more severe bone destruction in hyperuricemic mice than in normouricemic mice. They concluded that obesity increases the risk of periodontal disease by increasing the production of uric acid mediated by gut dysbiosis [93].

### 4.2. Other Disease Model Mice with Ligature-Induced Periodontitis

Some studies have evaluated the effects of periodontitis on systemic diseases. Periodontitis was induced in both WT and 5xFAD mice, a murine model of Alzheimer’s disease. Ligature-induced periodontitis increased the levels of Iba1-immunostained microglia in WT mice, while an increase in the level of insoluble Aβ42 was observed in 5xFAD mice. A decline in Iba1 in the proximity of Aβ plaques in 5xFAD mice with ligature-induced periodontitis compared to those without periodontitis suggested a periodontal disease-induced decrease in plaque-associated microglia. In addition, periodontitis reduced IL-6, MCP-1, granulocyte-macrophage colony-stimulating factor, and interferon-γ (IFN-γ) expression in the brains of WT mice and reduced IL-10 expression in 5xFAD mice. These data demonstrated that periodontitis increased neuroinflammation in WT mice and disrupted the neuroinflammatory response in 5xFAD mice, suggesting that microglia are central to the association between periodontal disease and Alzheimer’s disease [94]. Another study using AβPP/PS1 double transgenic mice showed that periodontitis exacerbated learning and memory impairment and augmented amyloid-β and neuroinflammatory responses. Ligature-induced periodontitis with *P. gingivalis* LPS injection into the periodontal tissue caused cognitive impairment and a significant reduction in the number of neurons [95].

Periodontitis is regarded as a prominent risk factor for cardiovascular disease and stroke in epidemiological studies [96,97]. Apolipoprotein E (ApoE) knockout mice fed a high-fat diet were subjected to periodontitis by ligation of silk ligature with or without *P. gingivalis* LPS injection at the ligated sites. Mice that underwent ligation with or without *P. gingivalis* LPS showed severe periodontitis, systemic inflammation, and aortic plaque formation. The magnitude of systemic inflammation and aortic plaque formation was notably greater in the ligated mice injected with *P. gingivalis* LPS [98]. However, ligature-induced periodontitis with an intravenous injection of LPS from *P. gingivalis* did not affect acute stroke pathology in terms of severity, determined primarily by infarct volume, despite the observation of elevated systemic inflammation. Ligature-induced periodontitis with repeated LPS challenge did not alter infarct volume, blood-brain barrier (BBB) breakdown, or systemic inflammation after experimental stroke [99].

Whether pre-existing periodontal disease condition exacerbates, or removal of such conditions ameliorates, medication-related osteonecrosis of the jaw (MRONJ) development after tooth extraction was investigated. A combination of ligature-induced periodontitis and tooth extraction mouse models with the administration of zoledronic acid or an anti-receptor activator of nuclear factor-kappa Β ligand (RANKL) Ab provided experimental evidence that a pre-existing pathologic inflammatory condition exacerbates ONJ development after tooth extraction in mice [100]. Fibrillin-1 insufficiency in Marfan syndrome (MFS) leads to structural weakness, which causes various tissue disorders, including cardiovascular and periodontal diseases. Ligature-induced periodontitis in fbn-1-deficient mice (fbn-1c1039G/+ mice) with MFS showed slower wound healing compared to WT mice, but periodontal tissue destruction did not differ between these mice [101]. In addition, ligature-induced periodontitis in the *Phex* gene-null mutant phenotype (Hyp^−/0^) showed a major X-linked hypophosphatemia phenotype in oral mineralized tissues, consistent with variations in patient susceptibility to periodontal disorders. Bone and cementum mineralization appeared to be similarly disturbed, where the hypomineralized pericellular matrix surrounded cells, and the protein osteopontin accumulated in a tissue-specific manner, most notably in the perilacunar matrix surrounding osteocytes [102].

The effect of periodontitis on the development of asthma was investigated. Ligature-induced periodontitis reduced the total number of cells in the bronchoalveolar lavage in a mouse model of asthma [103]. Furthermore, periodontitis decreased macrophages, TNF-α, and INF-γ expression in a mouse model of chronic obstructive pulmonary disease (COPD). Periodontitis may influence the course of Th1 profile cells, cytokines, and pulmonary alterations [104].

Many studies investigating the relationship between periodontal disease and pregnancy have concluded that periodontal disease is a risk factor for preterm and low birth weight [105,106,107]. The pregnant mice were ligated at day 8 of gestation, and 10^9^ CFU of *P. gingivalis* in 2% carboxymethylcellulose was administered every other day. Pregnant mice developed more severe ABL and showed decreased expression of forkhead box P3 (FOXP3), transforming growth factor-beta (TGF-β), cytotoxic T-lymphocyte-associated protein 4 (CTLA-4), and CD28 mRNA in gingival tissue. Furthermore, a lower number of regulatory T cells (Tregs) were present in the cervical lymph nodes of pregnant periodontitis mice [108]. Another study demonstrated that periodontitis could be a risk factor for systemic bone loss, especially in postmenopausal women. Periodontitis was induced by ligation with *P. gingivalis* infection in mice that underwent ovariectomy. Periodontitis and ovariectomy induced a significantly higher femoral and mandibular bone loss than periodontitis or ovariectomy alone. In addition, mice with periodontitis induced by ovariectomy showed significantly higher serum levels of TNF-α than mice that underwent ovariectomy alone [109]. Ligature-induced periodontitis with *P. gingivalis* infection also showed aggravation of age-related macular degeneration. An increase in mRNA expression related to oxidative stress, angiogenesis, and pro-inflammatory mediators in the retinae was observed, whereas antioxidant and anti-inflammatory-related gene expression was notably decreased [110]. Periodontitis increased mRNA expression of TNF-α and IL-1β in the kidneys. Mice ligated with unilateral ureteral ligation showed an increased renal inflammatory response without showing a significant influence on renal interstitial fibrosis or renal function [111].

### 4.3. Summary

The contents of this section are presented in Table 2. Ligature-induced periodontitis mouse models are useful for evaluating the effect of periodontitis on many kinds of systemic diseases. This model can clarify the underlying mechanisms and the relationship between periodontal disease and other systemic diseases.

## 5. Systemic Effects

Although periodontitis is considered a disease restricted to periodontal tissue when originally defined, a growing number of studies have indicated a relationship between periodontal inflammation and extraoral health in the past two decades [112]. However, in diabetes, for instance, most of the research has proven the deterioration of the underlying disease if it coexists with periodontitis, but it is challenging to clarify the causality between them due to the limitations of human studies. Thus, the ligature model, which realizes pure evaluation and ignores the influence of primary disease and incidence time nodes of periodontitis, is an ideal model for studying its systematic effect.

### 5.1. Effect on the Cardiovascular and Blood System

In a recent study by Ribeiro et al., on the influence of periodontitis on the cardiovascular system, a 4-0 sterile silk ligature was placed on the first molar of adult male Balb/c mice. The results showed increased levels of inflammatory cytokines in heart tissue, which led to sympathetic activity and cardiac dysfunction [113]. Two studies also reported changes in blood cell composition via the 5-0 ligature model using C57BL/6 mice. One study showed the augmentation of platelets and platelet-leucocyte interactions [114], and the other study reported anemia due to IL-6 induced hepcidin release in hepatic cells [115]. However, another study reported prominent inflammation in the liver and adipose tissue via long-term placement [116]. Despite inflammation in the liver, these findings suggested that the blood circulatory system is affected by periodontitis. A further normalized study should be performed to clarify the specific influencing mechanisms.

### 5.2. Effect on the Central Nervous System

As the influence on sympathetic activity has been reported [113], it is possible that periodontitis affects the whole nervous system due to blood circulation, including the brain. A previous study observed microgliosis and astrogliosis in the hippocampus during a range of 2–12 weeks of 5-0 ligature placement and indicated that the number of glial cells positively correlated with periodontal inflammation [117]. To explain gliosis, they proposed a hypothesis that inflammatory cytokines and toxins damaged the BBB during the pathogenesis of periodontitis. Subsequently, another research group applied a 7-0 silk ligature, proved the hypothesis, and showed that the permeability of the BBB increased when periodontitis was induced in similar mice [118]. Moreover, if ligature placement was prolonged to one year, the mice showed signs of cognitive impairment in addition to neural inflammation [119]. These findings paralleled those of clinical studies and provided objective evidence for the correlation between periodontitis and central nervous system disease.

### 5.3. Effect on the Intestinal Flora and Digestive System

Toxins and cytokines produced by bacteria and immune cells in the mouth, for which the dogma is considered to be the primary approach that periodontitis affects extraoral health, may limit the way we think. According to recent studies, the evidence linking the mouth and gut microbiota was presented as attributable to the ligature model, which induces periodontitis dispense with the extra bacterial components. Researchers used a 5-0 silk ligature to induce periodontitis for two weeks and found that the gut microbiota was utterly different [116]. A subsequent study confirmed the results and indicated that dysbiosis could disrupt intestinal barrier function [119]. To investigate the influencing mechanism of periodontitis-induced gut dysbiosis on the intestinal barrier, Kitamoto et al., induced periodontitis using the conventional method and found that the percentage of *Klebsiella* and *Enterobacter* species increased in both the mouth and gut [120]. They also proved that oral pathobiont-reactive T helper 17 (Th17) cells appeared in the gut. Based on their results, in addition to the inflammation caused by dysbiosis due to migration of oral microbiota, the oral pathobiont-reactive Th17 cells may also simultaneously migrate to the gut and exacerbate the inflammation. Although the theory currently applies only to the digestive tract, the migration of pathogenic T cells to other systems is worth exploring in the future because of the possibilities that may deepen the understanding of the systemic effects of periodontitis.

### 5.4. Summary

The contents of this section are summarized in Table 3. The ligature model can ignore the influence of primary disease and explore the effects of periodontitis on the whole body. Using this model, researchers have found that periodontitis possibly affects the central nervous system, cardiovascular system, blood system, and digestive system. Although it needs to be explored further, the theory of T cell migration may play a vital role in understanding the systemic effects of periodontitis, offering a new manner of thinking to periodontal medicine.

## 6. Immunology

Plaque biofilm is an initiating factor for periodontitis. Periodontal tissue can be directly destroyed by bacterial virulence factors, such as fimbriae and proteases [121,122]. In recent decades, an increasing number of studies have shown that the host immune response plays an integral role in the progression of periodontitis [123]. Inappropriate or excessive immune responses have been proven to be the main cause of periodontal tissue destruction during periodontitis [124,125]. The ligation-induced periodontitis model in mice is an important method of working on innate and acquired immunity against microbial infections. Compared with the *P. gingivalis* infection model in mice, the ligature-induced experimental periodontitis model may reflect broader pathogenesis in bone loss and inflammation [6,126].

### 6.1. Innate Immunity

Innate immunity, also known as natural immunity, is a series of natural defense functions that are activated at birth. Defense responses are quickly produced by the innate immune system when attacked by pathogenic microbes [127]. As part of the junction between the body and the outside environment, periodontal tissue also has its own innate immune response system. Epithelial tissue, saliva, gingival crevicular fluid, phagocytes, dendritic cells, and receptors identifying pathogenic microbes are all components of the periodontal innate immune system [16].

#### 6.1.1. Neutrophils

Neutrophils are important in innate immunity. Neutrophils account for approximately 30% of the total number of leukocytes circulating in murine bodies [128]. In a previous study, low levels of neutrophils were found in the oral cavity of healthy mice. Compared with neutrophils in other tissues, neutrophils in the oral cavity expressed more CD11a and CD66a. Ligature-induced periodontitis upregulated the expression of CD11b, implying that neutrophils could be transformed into a pro-inflammatory immunophenotype in the oral cavity of healthy and inflamed mice [129].

Normally, only mature neutrophils are released from the bone marrow. When inflammation occurs, neutrophils are recruited to regulate the inflammatory microenvironment under the activation of chemokines [130]. In a mouse model of periodontitis caused by ligation, PMNs were found to be elevated in the gingiva and bone marrow. The study continued to induce acute peritonitis in a mouse model of periodontitis and found that the number of PMNs in the blood and colon of mice with induced periodontitis and peritonitis increased more than that in mice with acute peritonitis alone. These results proved that periodontitis potentially impacts systemic inflammatory diseases. PMNs, which are generated inside the bone marrow due to periodontitis, could be recruited in the second inflammation site to influence the intensity of the innate immune response [11]. In another study, an anti-Ly6G antibody was used to deplete neutrophils in ligature-induced periodontitis. On days three and seven of the experiment, CD11b^+^ Ly6G^+^ neutrophils in the blood and gingiva, alveolar bone loss, and RANKL^+^ cells in the gingival of mice with experimental periodontitis were higher than those in the control group. After administration of the anti-Ly6G antibody, the number of Ly6G^+^ neutrophils and RANKL^+^ cells decreased in anti-Ly6G Ab-treated periodontitis mice on day three. However, on day seven, although the number of Ly6G^+^ neutrophils decreased significantly, the number of RANKL^+^ cells did not decrease. This research suggested that RANKL expression in periodontal tissue may be induced by neutrophils, leading to the early formation of osteoclasts [131]. The exosomes of neutrophils can also directly exert antibacterial effects [132]; the decrease in antibacterial activity caused by chronic neutrophil deficiency can also lead to an increase in bone loss [131].

In addition, in chronic periodontitis, the elevated production of reactive oxygen species by polymorphonuclear neutrophils exacerbates the destruction of periodontal tissue [41]. In the *Nrf*^−/−^ mouse model of ligation-induced periodontitis, an increase in 8-OHdG–positive cells and more severe alveolar bone loss and breakdown of periodontal soft tissue were found. Interestingly, compared with bone resorption, the damage caused by ROS in periodontal soft tissues is more significant [133].

#### 6.1.2. Macrophages

In addition to anti-inflammatory effects, macrophages can also aggravate the loss of bone and periodontal tissue by differentiating into M1 phenotypes with pro-inflammatory effects and osteoclasts. In macrophage-specific *Act1* expression downregulated (anti-*Act1* antisense oligonucleotides inserted) mice with ligature-induced periodontics, infiltration of macrophages in periodontal tissue and polarization of M1 macrophages increased. Micro-CT and histological tissue sections also showed that the downregulation of *Act1* expression leads to a higher degree of bone and tissue destruction with an increase in osteoclasts [134]. After treatment with C-C motif chemokine ligand 2 (CCL2), which can promote the migration of M0 or M2 phenotype macrophages to the inflamed site and locally induce M2 phenotype polarization, the loss of alveolar bone and the formation of osteoclasts in the ligation area were significantly reduced [135]. Surprisingly, M1 macrophages were also shown to inhibit RANKL-induced osteoclastogenesis in vivo. The mice that received M0 or M2 macrophage transfer showed significant bone loss compared to the mice that received M1 macrophage transfer, increasing the expression of TRAP-positive cells on the surface of the alveolar bone [136].

#### 6.1.3. Lymphoid Cells

Innate lymphocytes (ILCs) include three subtypes: ILC1s, which mainly express interferon-γ (IFN-γ); ILC2s, which mainly produce type 2 cytokines such as IL-5, IL-9, and IL-13; and ILC3s, which mainly produce the pro-inflammatory cytokines IL-22 and/or IL-17 [137]. AMP-activated protein kinase (AMPK) is a potential modulator or inhibitor of ILCs. In a previous study, all types of ILCs were found in the gingival tissue of healthy mice and mice with periodontitis. Although the quantity of the three subtypes of ILCs increased under periodontitis stimulation in *Prkaa1* (gene coding AMPK subunit α1) KO mice, the amount of ILC2s seemed to be the most affected. IL-33, an endogenous stimulator of cytokine expression by ILC2s, increased in both WT and *Prkaa1* KO mice, while IL-5 and IL-13 increased significantly only in *Prkaa1* KO mice [138].

#### 6.1.4. Solitary Chemosensory Cells

In a recent study, solitary chemosensory cells (SCC) were detected in the epithelial junction of mice. Such cells can detect bacteria and activate innate immunity through bitter Tas2r receptors and taste signal transduction. Induction of periodontitis by ligation in mice lacking SCC function showed more severe alveolar bone loss and gingival inflammatory infiltration. Meanwhile, colonization of the ligation sites was characterized by high bacterial load, low diversity, and high pathogen levels. In addition, local activation of SCC function enhanced expression of β-defensin-3 (Defb3) in the gingiva of mice to reduce bacterial load [139].

#### 6.1.5. Receptors

Innate immune recognition is based on the detection of microbial metabolism and gene products. In recent years, TLRs have been identified as the most critical immune receptors. There are 13 different types of TLRs in mammals that can specifically recognize a variety of microorganisms [140]. Among them, TLR2, TLR4, and TLR9 play important roles in the development of periodontitis.

Unlike periodontitis caused by *P. gingivalis* infection, ligation-induced periodontal disease is considered to cause alveolar bone loss in a TLR-independent manner. In *Tlr2* KO and *Tlr4* KO mice, the bone loss caused by ligation-induced periodontitis did not decrease. The mRNA expression of IL-1β and TNF-α in gingiva was also significantly increased, while the expression of IL-10 was greatly reduced in all types of mice [141]. In both *Tlr2* and *Tlr4* KO models, the expression of RANKL mRNA in the gingival tissue of ligation-induced experimental periodontitis was still significantly higher than that in the control group [126]. However, in a murine ligature-induced model of periodontitis, knockout of *Tlr9* reduced inflammatory infiltration of the gingival tissue and bone loss [142].

Inhibition of TLR signaling also seems to be a potential treatment option for periodontitis. Encoded by the *Tnfaip3* gene (TNF-α inducible protein 3), *A20*, as an important negative regulator of TLR signal transduction, can inhibit part of the NF-κB pathway by regulating inflammation [143]. The expression of A20 mRNA was primarily enhanced in the ligated tissues of *Tlr9* KO mice compared to the control group [142]. In *A20* haploid-deficient mice (*A20*^+/−^), more severe alveolar bone loss and soft tissue inflammatory cell infiltration at the ligature site were observed [144].

In addition to TLRs, a study also showed that human chemokine-like receptor 1 (ChemR23) could prevent bone loss. In *Cmklr1* (ChemR23 coding gene) transgenic mice, alveolar bone resorption caused by ligation was downregulated [145].

#### 6.1.6. Complements

In periodontitis, there is evidence that the C5aR complement plays an important role in the colonization of microorganisms in periodontal tissues and inflammation-mediated bone loss. After local treatment with a C5aR (CD88) antagonist, the expression of pro-inflammatory factors such as IL-6, TNF, and G-CSF in the ligation area was significantly inhibited [146]. Central complement C3 was found to play a vital role in maintaining the number of dysbiotic microbiota and destroying inflammation in periodontics. In *C3*-deficient mice, the loss of alveolar bone due to ligation was significantly induced compared to WT mice [147].

### 6.2. Acquired Immunity

Acquired immunity includes B cell-mediated humoral immunity and T cell-mediated cellular immunity. B cells differentiate into plasma cells with specific antibodies and memory B cells through direct activation of antigens [148]. Multiple effector T cells differentiated from T cells participate in the immune response through recognition-binding-activation [149]. Th17 and B10 cells appear to play an irreplaceable role in the immune regulation of periodontitis.

#### 6.2.1. T Cells and IL-17

The role of T cells in periodontal disease is still challenging to clarify. Th17, which can secrete IL-17, is a newly discovered subset of T cells [150]. Th17 cells transformed from FOXP3^+^ T cells mediated by IL-6 were reported to induce the expression of RANKL in osteoblasts and periodontal ligament cells by producing IL-17, which can effectively protect the body from pathogens by mediating the loss of alveolar bone and tooth [151]. In a recent study, the expression of *Il17a* was moderated in the first two days after the periodontitis model was established but increased rapidly in the subsequent days. This study also suggested that anti-IL-17A Abs decreased IL-6 gene expression in gingival tissue of periodontitis-induced mice and suppressed alveolar bone loss and osteoclastic activity; hence, IL-17A Abs might be an effective treatment for periodontitis [152]. In a recent study, IL-10 was reported to act as a negative feedback regulator to inhibit excessive IL-17 activity. In the *IL10*^−/−^ ligation model, *Il17* transcription, osteoclast differentiation activity, and polarization of M1 macrophages were significantly increased [153].

With the loss of alveolar bone, an increase in IL-17 secretion was also observed in *Bdkrb1* knockout mice. This study suggested that the kinin B1 receptor may be a modulator of T cells in the pathogenesis of periodontitis [154].

#### 6.2.2. B Cells and IL-10

B cells have been reported to contribute to late-onset periodontitis lesions. In the B-cell ablation mouse model, the bone loss at the later stage of ligation placement was significantly less than that in WT mice. After using proliferation-inducing ligand (APRIL) and B-lymphocyte stimulator (BLyS) antibodies as B-cell inhibitors, significant inhibition of B cells in gingival tissue and alveolar bone resorption were observed [155]. B10 cells are regulatory B cells that can produce the anti-inflammatory factor IL-10, which has been reported to inhibit the excessive inflammatory response in periodontitis immunomodulation, and seem to be potential target cells for periodontitis treatment. Cytidine-phosphate-guanosine oligodeoxynucleotide (CpG ODNs) and CD40 ligand (CD40L), which are agonists of *Tlr9*, suppressed periodontal inflammation and bone loss in the alveolus of WT mice with induced-periodontitis [156]. However, CpG ODNs and CD40L upregulated IL-10 protein expression and downregulated inflammation-related mRNA expression in both WT and TLR9 knockout mice, irrespective of TLR9 [157]. Using co-stimulatory molecules (CD40L, IL-21, and anti-Tim-1 mAb) for B10 cells in ligation-induced periodontitis mice could also upregulate the mRNA expression of gingival IL-10 while decreasing the expression of RANKL [158].

### 6.3. Cytokines, Molecules, and Genes

In addition to immune cells, cytokine networks and some molecules are indispensable for regulating immunity. A previous study suggested that TGF-β1, expressed by junctional epithelium, worked as a key anti-inflammatory cytokine in periodontitis modulation. Thus, IL-6, an activator of TGF-β1 activation, was believed to have a protective effect against periodontitis. In the ligated integrin β6-null mice, more severe inflammatory infiltration and bone resorption were observed. The results showed that the absence of integrin αvβ6 in the junctional epithelium could lead to the downregulation of the *Aim2* inflammasome and anti-inflammatory *Il10* [159]. Macrophage migration inhibitory factor (MIF) is a cytokine with pro-inflammatory effects and is an important regulator of endogenous glucocorticoids. In *Mif*-deficient mice, the compensatory upregulation of IL-6 and the downregulation of corticosterone levels were observed. However, the release of matrix metalloproteinase-2 (MMP-2) in the periodontal tissue of mice did not seem to be affected by MIF [160]. Vascular endothelial growth factor C (VEGFC), the main growth factor of lymphatic vessels, was found to be upregulated in tissues affected by periodontitis. Although overexpression of *Vegfc* enlarged lymphatic vessels in the oral cavity, the hyperplastic lymphatic vessels did not seem to play a protective role against the progression of periodontitis [161]. Compared to WT mice, less alveolar bone loss, osteoclast formation, and expression of TNF-α were observed in ligation-induced periodontitis in *cot/tp12*-deficient (*cot/tp12*^−/−^) mice. This study indicated that Cot/Tp12 was involved in alveolar bone loss caused by periodontitis [12]. MicroRNA-21 has also been reported to be a feedback mechanism in the inflammation regulation process of periodontitis. After *Mir21a* was knocked out from the mouse model, ligation caused the upregulation of IL-6, TNF-α, IL-1β, and RANKL in the gingival tissue and increased alveolar bone loss [162]. Under the irritation of periodontitis, human antigen-R (HuR) maintains the stability of IL-6 by binding to the 3′untranslated region (3′ UTR) of IL-6 mRNA. In a murine periodontitis model induced by ligation, a HuR inhibitor could prevent bone resorption by reducing the level of IL-6 [163]. Glucose-dependent insulinotropic polypeptide (GIP) has been reported to be involved in inflammation via the GIP receptor (GIPR) [164], increasing fat deposition in adipose tissue and promoting bone formation [165,166]. A previous study reported that *Gipr* knockout caused severe periodontitis with increased inflammatory cell infiltration in an experimental periodontitis mouse model. *Gipr*-knockout mice with ligature-induced periodontitis also showed a significant increase in the gene expression of gingival inflammatory cytokines, TNF-α, and inducible nitric oxide synthase (iNOS), as compared to WT mice with experimental periodontitis. These results suggested that GIP exerts anti-inflammatory effects on periodontitis [167]. In another study, protein tyrosine phosphatase-α (PTPα) was believed to promote extracellular signal-regulated kinase (ERK) activation and MMP-3 expression, thereby affecting IL-1β and inducing the degradation of periodontal connective tissue [168].

### 6.4. Summary

The contents of this section are summarized in Table 4, Table 5 and Table 6. In immunological research, the ligation-induced periodontitis mouse model has been widely used. It is appropriate to use transgenic or gene knockout murine models to study the role of specific genes in the process of periodontitis.

## 7. Microbiology

### 7.1. Oral and Gut Microbiota in Ligature-Induced Periodontitis Model

In recent years, three studies have investigated the oral microbiota of periodontitis mice using 16S rDNA amplicon sequencing [119,169,170]. One study investigated the microbiota attached to ligatures in a mouse model of cherubism and reported that the most predominant bacteria in the ligatures were Pasteurellales, followed by Lactobacillales [169]. On the other hand, a study investigating the microbiota of saliva reported that Proteobacteria was the most abundant phylum detected in both ligature-induced periodontitis mice and mice without periodontitis [119]. This study also reported that the phyla Firmicutes, Actinobacteria, Acidobacteria, Chloroflexi, Oxyphotobacteria, Gemmatimonadetes, Thaumarchaeota, and Nitrospirae were significantly more abundant in ligature-induced periodontitis mice than in control mice, whereas Proteobacteria was significantly less abundant in ligature-induced periodontitis mice than in control mice. In terms of diversity, alpha-diversity, which captures the diversity of species within the sites, was significantly higher in ligature-induced periodontitis mice than in control mice. In addition to the analysis to identify bacterial species using 16S rDNA amplicon sequencing, there have also been studies on the oral microbiome of mice by counting viable bacteria and by quantitative analysis of bacteria using real-time PCR on gingival tissue surrounding the ligature [171,172].

Several studies have examined the gut microbiota of ligature-induced periodontitis mice [119,173,174]. One study reported that *Parabacteroides* and Desulfovibrionaceae increased and several butyrate-producing bacteria decreased significantly in the gut microbiota of ligature-induced periodontitis mice compared to control mice, and alpha-diversity of the gut microbiota in the periodontitis mice was significantly reduced compared to that of control mice [174]. In addition to the study that investigated the bacterial composition of the gut microbiota by 16S rDNA sequencing, the Phylogenetic Investigation of Communities by Reconstruction of Unobserved States (PICRUSt) software was used to predict metagenome function from the 16S rDNA data [173]. PICRUSt metagenome predictions have limitations in terms of the level of functional classification. However, to the best of our knowledge, no previous studies have investigated the gut microbiota of ligature-induced periodontitis using shotgun metagenomic sequencing.

The taxonomic composition of the oral and gut microbiota has been studied to investigate the relationship between the oral and gut microbiota of ligature-induced periodontitis [119]. A previous study reported that the oral and gut microbiota of ligature-induced periodontitis mice exhibited a significant increase in alpha-diversity indices compared to control mice. Additionally, the non-metric multidimensional scaling analysis showed that the microbial composition in the ligature-induced periodontitis mice clustered separately from those in the control group in both saliva and feces. The results showed that the compositions of the oral and gut microbiota were different, and Bacteroidetes accounted for nearly 50% of the gut microbiota in a mouse model of periodontitis, whereas most of the oral bacteria were Proteobacteria, with a ratio of approximately 75% at the phylum level.

### 7.2. Summary

The contents of this section are presented in Table 7. As a summary of the oral and gut microbiota of ligature-induced periodontitis mice, ligature induction can cause dysbiosis in the oral and gut microbiota, and the oral microbial composition is different from that of the gut.

## 8. Ligature-Induced Periodontitis Models with Bacterial Factors

While a number of studies have been reported using a periodontitis model induced by ligation alone, several studies have been reported using periodontitis models induced by ligation with the addition of bacteriological factors [9,10,175,176,177,178,179,180,181,182,183,184,185,186,187,188,189,190,191,192,193,194,195]. In this paragraph, we have reviewed the additional factors that enhance periodontal conditions and each characteristic.

### 8.1. Periodontitis Model Induced by Ligature Inoculated with P. gingivalis

Many studies have used the periodontitis model by placing ligatures inoculated with *P. gingivalis* around the teeth of the mice to induce massive local accumulation of periodontal pathogens [10,177,178,179,180,181,182,183,184,185,186,187,188,190,191,194,195]. In a representative study using this model, Li and Amar [188] provided evidence that the mouse periodontal model involving a ligature inoculated with *P. gingivalis* exhibited a more adequate inflammatory response and greater periodontal tissue breakdown than the control group, which induced periodontitis by ligature alone. Additionally, the results from a similar study revealed that *P. gingivalis*-infected mice showed significant bone loss at the sites where the ligatures were tied for 13 to 15 weeks compared to the ligature-induced periodontitis model without infection [194].

### 8.2. Periodontitis Model Induced by Ligation and Lipopolysaccharide of P. gingivalis

Two studies using the ligature-induced periodontitis model added periodontal pathogenicity by injection of LPS from *P. gingivalis* [9,176]. A previous study mentioned that the injection of *P. gingivalis* LPS in gingiva directly could avoid inducing various inflammatory changes linked to periodontitis-associated systemic disorders, instead of using ligature-soaked *P. gingivalis* [176]. Another study investigated the relationship between periodontitis and aging by comparing the ligature alone and the ligature plus *P. gingivalis* LPS model [9]. The results showed that young mice with periodontitis induced by ligation and *P. gingivalis* LPS had significantly elevated secretion of senescence-associated secretory phenotype (SASP) markers, including pro-inflammatory cytokines TNF-α, IL-6, and IL-1β, as well as osteoclastogenic RANKL, and a higher number of osteoclasts compared to periodontitis mice induced by ligature alone. Although there are a limited number of studies, these two reports suggest some advantages of using a mouse model of periodontitis with ligation and *P. gingivalis* LPS.

### 8.3. Periodontitis Model Induced by Ligation and Oral Gavage of P. gingivalis

Aside from the ligature-induced periodontitis model, there is also a common method that induces periodontitis in mice by oral gavage with *P. gingivalis* [6,196,197,198]. However, it was reported that the ligature-induced periodontitis model was an effective approach to induce periodontal inflammation similar to human periodontitis, whereas periodontitis models induced by oral gavage alone were not effective in stimulating tissue destruction [199]. Therefore, to investigate the difference in periodontal tissue destruction after local and/or systemic dysbiosis induction, Palioto et al., conducted a study comparing the three periodontitis models: ligature, *P. gingivalis* gavage, and ligature with *P. gingivalis* gavage, i.e., local, systemic, and local and systemic dysbiosis, respectively [189]. The results showed that all three groups induced significant bone loss compared to the sham group, especially in the *P. gingivalis* gavage and *P. gingivalis* gavage with ligature groups. With regard to the systemic inflammatory markers in these periodontitis models, the ligature and *P. gingivalis* gavage groups induced significantly higher serum cytokines. Surprisingly, *P. gingivalis* gavage + ligature mice induced significantly fewer markers. They also revealed that the number of inflammatory markers on the surface of alveolar bone and within the gingiva in the ligature with the *P. gingivalis* gavage group were higher than those in the ligature group alone, suggesting that epigenetic and inflammatory markers were elevated in the periodontium of mice treated with both ligature and *P. gingivalis* gavage. Additionally, gut tissue dissected from each periodontitis model was analyzed to examine alterations in gut inflammation. The inflammatory and epigenetic markers in the epithelial tissue of the gut in the oral gavage only group were higher than those in the ligature groups. These findings suggested that the oral gavage model might result in elevated systemic inflammation compared to the ligature model. They summarized that oral gavage or a combination of oral gavage and ligature-induction might be a good strategy for studying inflammatory markers and epigenetic changes in experimental periodontal disease and better for modeling human periodontal disease compared to the simple ligature model. In addition to the aforementioned methods, there is another method to promote periodontitis by orally administering *P. gingivalis* to a ligature-induced periodontitis model; however, few studies have used this method, and it may not be considered to be a common way to induce periodontitis [175,192].

### 8.4. Ligature-Induced Periodontitis Model Affected by Smoking Factors

One study aimed to examine the effect of smoking on alveolar bone destruction in a ligature-induced periodontitis model injected with nicotine and cigarette smoke condensate, which are the major constituents of cigarette smoke [200]. This study indicated that the ligature-induced periodontitis model was useful for elucidating the pathogenesis of cigarette-smoking-related periodontal diseases.

### 8.5. Summary

The contents of this section are presented in Table 8. Most studies on the enhancement of periodontal inflammation in ligature-induced periodontitis mice were based on the use of *P. gingivalis*. In particular, the method of placing ligatures inoculated with *P. gingivalis* was a common way to induce the progression of periodontitis. Several studies have been conducted with a ligature-induced periodontitis model with *P. gingivalis* LPS or oral administration of *P. gingivalis* by gavage or feeding. However, the most suitable method to develop periodontitis in the ligature-induced periodontitis model remains controversial.

## 9. New Treatment Methods

Recently, a ligature-induced periodontitis mouse model has been used in many studies concerning the effects of various novel treatments on periodontitis.

### 9.1. Effects of Systemic Antibiotics

Systemic antibiotics can provide great benefits in the treatment of periodontal disease. Systemic antibiotic therapy can enhance the effect of mechanical debridement in overcoming periodontal infections, resulting in clinical improvements, such as a decrease in bleeding on probing, reduction in pocket depth, and gain in clinical attachment level [201,202]. Several reports have demonstrated the effects of systemic antibiotics in a ligature-induced periodontitis mouse model [203,204,205].

A previous study reported that systemic antibiotics were effective in reducing bone loss caused by orthodontic force-aggravated periodontitis in ligated mice. After systemic antibiotic administration, there were reductions in periodontal bone loss and gingival mRNA and the protein RANKL/OPG expression ratio [203].

Macrolides have immunomodulatory effects in addition to their antimicrobial activity [206], and they are used as systemic antibiotics for periodontal treatment. However, their immunomodulatory mechanisms are not well understood. Maekawa et al., investigated the immunomodulatory effects of macrolides in a murine ligature-induced periodontitis model, focusing on developmental endothelial locus-1 (DEL-1) [204]. DEL-1 is a local homeostatic factor that regulates the initiation and resolution of inflammation, inhibiting neutrophil recruitment [207,208]. It was revealed that erythromycin (ERM), a 14-membered macrolide, significantly suppressed inflammation and inhibited bone loss in ligated mice, as compared to the control group and other antibiotics groups (josamycin and penicillin). Mice treated with ERM showed a significant decrease in neutrophil infiltration in the gingival tissue, downregulation of IL-17 and IL-6 mRNA expression, and upregulation of DEL-1 and IL-10 mRNA expression. On the other hand, ERM treatment failed to suppress bone loss and neutrophil migration or regulate cytokines in *Del1*-deficient mice with ligature-induced periodontitis. It also became clear that ERM exerts immunomodulatory effects by activating JAK2 and PI3K/AKT signaling pathways, which leads to activation of DEL-1 expression and reversal of IL-17-induced inhibition of DEL-1 expression [204]. ERM has also been reported to affect osteoclasts and alveolar bone resorption via DEL-1 induction in ligated mice. Treatment with ERM inhibited alveolar bone loss by increasing *Del1* expression and decreasing the expression of osteoclast-related factors, such as a nuclear factor of activated T cells (*Nfatc1*), a receptor activator of nuclear factor-κB (*Tnfrsf11a*), acid phosphatase 5 (*Acp5*), and cathepsin K (*Ctsk*) [205].

### 9.2. Effects of Peptides or Proteins

The anti-inflammatory functions of some peptides or proteins in periodontitis have attracted attention. The effects of such peptides, polypeptides, or proteins have been investigated in a ligature-induced periodontitis mouse model.

DC-STAMP, originally identified in dendritic cells (DCs), plays a key role in osteoclast cell fusion [209]. DC-STAMP mRNA expression is reportedly increased in patients with periodontitis [210]. The effects of DC-STAMP in periodontitis were evaluated using an anti-DC-STAMP-monoclonal antibody (mAb) in mice with ligature-induced periodontitis. Local injection and systemic administration of anti-DC-STAMP-mAb significantly downregulated the alveolar bone loss that occurred in ligature-induced periodontitis via upregulation of osteoclastogenesis. Anti-DC-STAMP-mAb did not affect the expression of TNF-α, Il-1β, and RANKL, which were elevated by ligature-induced periodontitis. These results indicated the role of DC-STAMP in promoting local osteoclast cell fusion without affecting adaptive immune responses to oral bacteria [211].

The wingless/integrase-1 (Wnt) signaling pathway plays an important role in cell migration, proliferation, and immunomodulation. Abundant expression of Wnt5a is associated with several human pathologies, including cancer, fibrosis, and inflammation [212]. In the gingiva of chronic periodontitis, the expression of Wnt5a mRNA is reported to increase [213]. Maekawa et al., reported that there was a novel interrelationship between Wnt5a and one of its functional antagonists, secreted frizzled-related protein 5 (sFRP5), in periodontal health and disease. Mice with ligature-induced periodontitis showed a high expression of Wnt5a and low expression of sFRP5. Local sFRP5 administration reversed this profile and significantly inhibited inflammation and bone absorption, correlating with the decreased number of osteoclasts in bone tissue. It was shown that sFRP5 blocked experimental periodontal inflammation and bone loss, indicating the possibility of using the Wnt5a/sFRP5 ratio as a periodontitis biomarker [214].

Food-derived peptides have been reported to have a wide range of activities, including antibacterial effects, blood-pressure-lowering effects, antioxidant activities, and cytoprotective or immunomodulatory effects [215,216]; however, little is known about their effects on inflammation and bone resorption in periodontitis. Several researchers have investigated the effects of bioactive peptides derived from foods on periodontitis [217,218]. Local treatment with rice endosperm protein (REP) 9 and 11 significantly inhibited the activity of inflammatory and osteoclast-related molecules and significantly decreased bone resorption in a ligature-induced periodontitis mouse model [217]. Another peptide from rice, Amyl-1-18, also significantly prevented alveolar bone destruction in mice with ligature-induced periodontitis via suppression of LPS-induced inflammatory cytokine production. These results suggested that the Amyl-1-18 peptide has anti-inflammatory properties against LPS [218].

### 9.3. Effects of Natural and Synthetic Compounds

Several natural and synthetic compounds have been investigated for their potential as therapeutic agents in periodontal disease. A previous study focused on the effects of reveromycin A (RMA) in a mouse model of ligature-induced periodontitis [219]. RMA is an acidic compound produced by *Streptomyces reveromyceticus* and inhibits bone resorption by inducing apoptosis specifically in osteoclasts [220]. RMA treatment significantly decreased osteoclasts, alveolar bone loss, attachment loss, and inflammatory cytokine expression, especially in osteoprotegerin (OPG, coded by *Tnfrsf11b*) knockout mice with ligature-induced periodontitis. It has been suggested that RMA prevents alveolar bone resorption and inflammation in periodontal tissue [219].

Myricetin, a natural flavonoid compound, is widespread among plants, including vegetables, fruits, tea, and medicinal herbs [221]. Previous studies have reported that myricetin protects osteoblasts against apoptotic cell death induced by inflammatory cytokines, induces osteoblast differentiation, and inhibits osteoclastogenesis [222,223,224,225]. The effects of myricetin on periodontitis in an ovariectomized (OVX) mouse model were investigated, and it became clear that myricetin inhibited osteoclast formation and prevented alveolar bone loss. This study strongly suggested that myricetin may be a potentially useful agent for the treatment of periodontitis [226].

Another study focused on a natural compound, oleanolic acid acetate (OAA), which is abundant in olive fruit and various legumes [227]. OAA is commonly used for the treatment of inflammatory diseases and various types of cancers [228,229]. This study revealed that OAA in the treatment of periodontitis induced bone formation and remodeling via proper modulation of osteoblasts, osteoclasts, and inflammation by regulating TGF-β and Wnt signaling [230].

Shogaols are the main bioactive compounds in dried ginger, among which 6-shogaol has been widely used to treat various ailments, including inflammation [231,232]. A study investigated the effects of 6-shogaol on osteoclast differentiation and bone resorption in a ligature-induced periodontitis mouse model. Administration of 6-shogaol prevented osteoclastogenesis and alveolar bone resorption caused by ligature-induced periodontitis, decreased the number of macrophages and neutrophils, and considerably downregulated the expression of IL-1β and TNF-α in periodontal tissue. These results confirmed that 6-shogaol had anti-osteoclastogenic effects and suggested its potential application as an anti-resorptive treatment in periodontitis [233].

Some studies have evaluated the potential of synthesized compounds as therapeutic agents for periodontitis. A novel pyrimidine derivative, OCLI-023, was examined for its effect on bone resorption in ligature-induced experimental periodontitis in mice. OCLI-023 significantly decreased the distance between the cementoenamel junction and alveolar bone crest and reduced the number of osteoclasts induced by periodontitis. OCLI-023 inhibited ligature-induced bone loss [234]. The effects of another synthesized compound, a novel benzamide-linked derivative (OCLI-070), were also investigated in mice with ligature-induced periodontitis. OCLI-070 inhibited osteoclast formation and differentiation, reduced *Nfatc1* and the expression of osteoclast-specific genes, and prevented bone resorption via the suppression of RANKL-mediated ERK and NF-κB signaling pathways [235]. A previous study tested another new anabolic compound, LLP2A-alendronate (LLP2A-Ale), as a possible agent for the treatment of periodontal disease. LLP2A-Ale has an affinity for both bone and mesenchymal stromal cells (MSCs). LLP2A-Ale has been reported to direct MSCs to the bone for new bone formation and increase bone strength [236,237]. Treatment with the novel compound stimulated alveolar bone formation and reversed bone loss caused by ligature-induced periodontitis. LLP2A-Ale also reduced the levels of periodontitis-induced circulating inflammatory cytokines. Treatment with a combination of MSCs and LLP2A-Ale further increased peripheral skeletal bone formation and bone mass. These results suggested that LLP2A-Ale treatment can be a novel therapeutic option for the treatment of bone resorption caused by periodontitis [238].

### 9.4. Effects of Extracts from Plants

The extracts from plants were used for periodontal treatment in some experiments. Boldine, an isoquinoline, decreased osteoclast numbers and the RANKL/OPG ratio in periodontal sites. In addition, the Th17-lymphocyte detection and response were reduced, and the Treg-lymphocyte detection and response were increased in periodontitis-affected tissues by boldine. These results indicated that administration of boldine in the ligature-induced periodontitis model modulated the Th17/Treg imbalance [239].

Some studies have employed resveratrol, a natural phenol. C57BLKS/J-*db/db* mice were used for the ligature-induced periodontitis model to elucidate the effects of resveratrol. Blood glucose levels were decreased, alveolar bone loss was ameliorated, and high levels of IL-1β, IL-6, IL-8, TNF-α, and TLR4 were suppressed by resveratrol in the gingival tissue of the murine model [240]. Additionally, resveratrol derivative-rich melinjo seed extract (MSE) decreased oxidative stress as demonstrated on immunohistochemistry. M-CSF/sRANKL-mediated osteoclast formation was inhibited, and osteoclast activity on the periodontal side was downregulated by MSE [241]. These reports suggested that resveratrol may be a valid treatment for periodontitis.

Another agent extracted from plants, glycyrrhizic acid, which is the main active compound in licorice, has been reported to exert anti-inflammatory effects. The effects of glycyrrhizic acid on a combination of *Porphyromonas gulae* and ligature placement in a diabetic model mice were also examined. Glycyrrhizic acid suppressed the release of inflammatory cytokines, high mobility group box 1, and a receptor for AGE, which were increased by ligature/*P. gulae* at the mRNA level in gingival tissue and the protein level in the serum of diabetic mice [47].

Another study reported that systemic administration of green tea relieved alveolar bone loss and reduced the number of inflammatory cells and osteoclasts [242]. Thus, treatment with galenica showed some possibility of improving periodontitis.

### 9.5. Effects of Treatment Instrumentation

Non-surgical periodontal treatment (NSPT), such as scaling and root planing, is a commonly employed form of mechanical removal of bacterial pathogens in periodontal therapy. NSPT was applied to *ApoE*^−/−^ mice with ligature-induced periodontitis, resulting in the prevention of alveolar bone loss, improvement of lipid profile, and inhibition of systemic inflammation with reduced plasma IL-6 levels. NSPT treatment in *ApoE*^−/−^ mice also showed reduced inflammation in the arterial wall, less vascular cell adhesion molecule-1 expression, and less macrophage adhesion. This indicated that alveolar bone levels were reduced, and systemic inflammation was inhibited by NSPT [243].

Low-intensity pulsed ultrasound (LIPUS) was applied in a ligature-induced mouse model to investigate its effects on oxidative stress in periodontitis as a new therapeutic option for periodontal disease. LIPUS treatment alleviated alveolar bone homeostasis in periodontitis by downregulating oxidative stress via PI3K-Akt/nuclear factor erythroid 2-related factor (NRF2) signaling. The study revealed that NRF2 played a pivotal role in the protective effect exerted by LIPUS against ligature-induced experimental periodontitis [244].

### 9.6. Effects of Antibody, Cell, Cytokine etc.

Many researchers have developed new inhibitors for periodontal treatment. For instance, treatment with JTE-013, which is known to inhibit sphingosine-1-phosphate receptor 2 (S1PR2), attenuated ligature-induced alveolar bone loss and reduced inflammation-related gene expression levels [245]. Administration of IMD-0354, a novel I kappa-B kinase (IKK) inhibitor, significantly suppressed linear bone resorption and RANKL gene expression levels in gingival tissue compared to the control group. The results indicated that the IKK inhibitor has a role in the suppression of RANKL gene expression via downregulation of NF-κB [246]. Additionally, previous studies have examined the effects of PF-3845, an inhibitor of fatty acid amide hydrolase (FAAH), and decitabine, which is a DNA methyltransferase inhibitor used to treat ligature-induced periodontitis. Bone loss and osteoclast differentiation in experimental periodontitis were inhibited by both pharmaceuticals. Medication of PF-3845 demonstrated anti-osteoclastogenic and anti-resorptive activities by suppressing the phosphorylation of rapidly accelerated fibrosarcoma (RAF), mitogen-activated protein kinase (MEK), ERK, and NF-κB inhibitor (IκBα) [247]. Decitabine inhibited osteoclastogenesis through the upregulation of anti-inflammatory cytokines via Krüppel-like factor-2 (KLF2)-dependent mechanisms; therefore, decitabine reduced bone loss in a mouse model of ligature-induced periodontitis [248].

Some studies have reported that therapeutic agents for systemic diseases are also effective medications for periodontitis. One of the studies used normal and diabetic mice with ligature-induced experimental periodontitis to evaluate the effects of aliskiren. Aliskiren is a renin inhibitor used as a medicine for hypertension. Renin was found to be related to periodontitis-induced bone loss and inflammatory responses in diabetic mice. Aliskiren treatment reduced gene expression of renin-angiotensin system (RAS) components in the gingival tissue of normal and diabetic mice. Administration of aliskiren also downregulated the gene expression of COL1A1, COL1A2, and fibronectin, which were upregulated by periodontitis. These results suggested the presence and functional role of local RAS in gingival tissue, exacerbating the inflammatory response, periodontal bone loss, and wound healing processes in both normal and diabetic animal groups [249]. Another study used *ApoE*^−/−^ mice with periodontitis to investigate the effects of rosuvastatin. Rosuvastatin, which has been applied for the treatment of atherosclerosis, inhibited TNF-α-induced osteoclast formation, endothelial cell phenotypic changes, foam cell formation, and the expression of CD47 and other oncogenes in arterial smooth muscle cells related to atherosclerosis. This study indicated that rosuvastatin reduced local inflammation and ligature-induced bone loss, decreased systemic inflammation caused by periodontitis, and prevented the exacerbation of atherosclerosis induced by periodontitis in *ApoE*^−/−^ mice [98].

Furthermore, various therapeutic agents have been used to treat periodontal disease in a ligature-induced periodontitis mouse model. Surface pre-reacted glass ionomer (S-PRG) elute, a component of composite resin, has been reported to prevent periodontitis, and its effect was evaluated in experimental periodontitis in ligated mice. Histological analysis clarified that the S-PRG elute suppressed the destruction of the collagen bundle in the periodontal ligament and inhibited the infiltration of inflammatory cells. Immunohistochemical analysis revealed that the number of infiltrating neutrophils and macrophages was significantly suppressed. These results showed that S-PRG released metal ions, which had the potential to prevent periodontitis [250]. Interestingly, one research group examined the treatment of anti-glycation agents (PTB) in a ligature-induced periodontitis model. They prepared four experimental groups: no periodontitis induction, ligature-induced experimental periodontitis (PR), experimental periodontitis plus hydrogel without PTB (PH), and experimental periodontitis plus hydrogel with PTB (PP). PTB significantly reduced periodontal bone levels (PBL) and inflammatory cell infiltration in PP during the induction phase. Moreover, PTB significantly decreased PBL and inflammatory cell infiltration, and significantly greater collagen deposition was observed in PP than in PR and PH at 4 and 14 days after ligature removal [251]. Some studies have reported the use of bone marrow stromal cells and MSCs as a treatment for periodontitis. In a study demonstrating the effects of bone marrow stromal cells (BMSCs), BMSCs were applied to the gingiva of the mesial and interdental papilla around the ligated molar. Micro-CT analysis revealed that alveolar bone loss around the ligated molars was significantly reduced in BMSC-treated mice compared to non-treated mice. Inflammatory infiltration was also decreased, and the number of TRAP-positive osteoclasts was notably increased in ligature-induced mice treated with BMSC [252]. In another study, the authors applied exosomes from TNF-α-treated human gingiva-derived MSCs (GMSCs) as a therapeutic agent. Local injection of GMSC-derived exosomes significantly decreased bone loss and the number of TRAP-positive osteoclasts, and these effects were further enhanced by preconditioning the GMSCs with TNF-α. Both exosomes with and without TNF-α reduced RANKL mRNA expression and significantly elevated OPG mRNA expression [253]. Nagai et al., evaluated the capacity of hydrogel-formulated inhibitors, including prolyl-4-hydroxylase (1,4-DPCA/hydrogel), to promote regeneration of bone loss due to periodontitis. Injection of 1,4-DPCA/hydrogel increased gingival hypoxia-inducible factor 1α (HIF-1α) protein levels and alveolar bone regeneration. The 1,4-DPCA/hydrogel-induced regeneration of alveolar bone was associated with enhanced expression of osteogenic genes, decreased expression of inflammation-related genes, and increased abundance of Tregs in the periodontal tissue [254].

### 9.7. Summary

The contents of this section are presented in Table 9. An experimental ligature-induced periodontitis model in mice can simulate pathological conditions of human periodontitis, including local and systemic inflammation and destruction of periodontal tissue. Therefore, this model is useful for examining the effects of various treatment methods and therapeutic agents on periodontal disease including reduction in bacteria and its pathogenic factor, anti-inflammation, and prevention of alveolar bone loss. Studies using this model can also observe the healing process of periodontitis and help to understand the underlying mechanisms.

## 10. Conclusions

**Figure 1:**
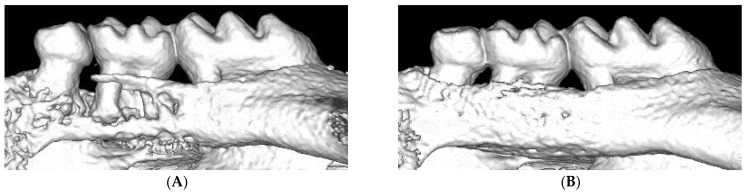
Healing of alveolar bone resorption without treatment in ligation-induced periodontitis in mice. A ligature was placed around the maxillary second molar in 10-week-old male C57BL/6 mice (wild-type, WT) for seven days. Micro-CT evaluation showed natural healing of alveolar bone loss after removal of ligature. (**A**) Before ligature removal (**B**) Seven days after ligature removal.

The ligature-induced periodontitis model in mice provides useful information on the pathogenesis and treatment of periodontal disease. Although the model cannot reflect all aspects of the mechanisms or causes of periodontitis in humans, the elicitation of significant bone loss is a major feature of this model. Therefore, this model is an ideal technique for studying the mechanism of periodontitis-induced alveolar bone resorption and the method of suppressing alveolar bone loss. Numerous studies have shown that the activation of osteoclasts and inflammatory factors, as well as inappropriate or excessive immune responses, are the main causes of periodontal tissue destruction in the progression of ligature-induced periodontitis in mice. Since many immunologic and cellular reagents have already been applied in murine models, it would also be appropriate to apply such models to study the regulation of specific genes or cells in periodontal tissue. In addition, ligation can be applied to other disease models in mice to induce periodontitis, making it possible to investigate the interaction between periodontal disease and other systemic diseases, such as diabetes, obesity, and Alzheimer’s disease. Furthermore, for microbial evaluation of periodontitis, placing a ligature inoculated with *P. gingivalis* in mice can better reflect the interaction between microbes and inflammation. Finally, it is worth noting that the bone loss induced by the murine model healed seven days after ligature removal without any treatment (Figure 1). As a result, ligature-induced periodontitis appears to be an acute periodontitis model. Although this model may not reflect long-term bone loss and inflammatory infiltration in chronic periodontitis, the ligature-induced experimental periodontitis model in mice is effective for exploring the molecular mechanisms of periodontitis.

## Figures and Tables

**Table 1:** Summary of periodontal tissue of ligature-induced periodontitis model.

Reference No.	Year Author	Periodontal Tissue	Main Findings
[27]	2019 Chen et al.	Gingiva	*Grhl2* KO impaired the expression of the junction proteins at the junctional epithelium and increased the alveolar bone loss in the ligature-induced periodontitis model.
[31]	2021 Guo et al.	Gingiva	CTHRC1 was highly expressed in GCF and gingival tissues of periodontitis patients and animal models.
[32]	2018 Movila et al.	Gingiva	Induced inflammation was observed in the gingiva, using a novel intravital endoscopic technology.
[35]	2020 Choi et al.	Gingiva	GRP-positive cells were mostly located at the oral epithelium of samples from experimental periodontitis model
[38]	2017 Okanobu et al.	Gingiva	A significant increase in the degree of gingival overgrowth and expansion of the connective tissue area was observed in cyclosporine A (CsA) and ligature-induced periodontal model mice, whereas cessation of CsA and antibiotic administration reduced gingival overgrowth.
[39]	2019 Bao et al.	Gingiva	The gingival tissue protein abundance was shown to be mainly dependent on the progression of periodontitis by unsupervised clustering analysis. Additionally, over-representation of innate immune regulation, signal transduction, and homeostasis processes was revealed by gene ontology enrichment analysis.
[40]	2019 Huang et al.	Gingiva	Ferritin was detected in the gingival epithelium and gingival connective tissue by immunochemical staining; the intensity of positive staining became significantly stronger along with the extent of inflammatory infiltration.
[43]	2016 Kataoka et al.	Gingiva	The ligature model was used to visualize the oxidative stress induced by experimental periodontitis. The Nrf2/antioxidant defense pathway was activated and could be visualized in Keap1-dependent oxidative stress detector-luciferase mouse model.
[46]	2018 Ishii et al.	Bone	Systemic administration of anti-OC-STAMP-mAb suppressed the expression of CD9 mRNA, but not DC-STAMP mRNA, in periodontal tissue. OC-STAMP partnered CD9 to promote periodontal destruction by upregulation of fusion during osteoclastogenesis.
[48]	2018 Yoshihara-Hirata et al.	Bone	Administration of an anti-HMGB1 neutralizing antibody in an experimental periodontitis model attenuated alveolar bone resorption and inflammatory cytokines.
[50]	2019 Fu et al.	Bone	A significant decrease in PTEN was observed in ligature-induced periodontitis model. PTEN could directly inhibit inflammation and bone loss via inhibiting the IL-1 and TNF-alpha pathway in periodontitis.
[51]	2021 Liu et al.	Bone	Knockout of NRAGE induced autophagy-related gene expression and accelerated bone destruction by increasing the activity and differentiation of osteoclasts.
[53]	2019 Bostanci et al.	Bone	TREM-1 regulates the IL-17A-RANKL/OPG axis and bone loss in experimental periodontitis.
[55]	2020 Wang et al.	Bone	SLIT2 expression was upregulated in periodontitis in both humans and mice, and a higher expression of SLIT2 accelerated the progression of periodontitis.
[57]	2020 Kittaka et al.	Bone	Ligature-induced alveolar bone loss in *Sh3bp2*-deficient mice was reduced compared to WT mice. SH3BP2/SYK signaling axis regulates inflammatory response, osteoclastogenesis, and osteoclast functions in periodontitis.
[59]	2019 Huang et al.	Bone	In vitro experiments revealed that FICZ pre-treatment reduced LPS-induced inflammation in periodontal ligament cells via increased phosphorylation of STAT3. FICZ promoted the mineralization of PDLCs via activation of the Wnt/β-catenin signaling pathway.
[61]	2018 Kim et al.	Bone	*P2rx5*-deficient mice had decreased ligature-induced bone loss compared to WT littermates. Gene expression analysis of gingival tissue of ligated mice showed that *Il1b*, *Il6*, *Il17a*, and *Tnfsf11* expression levels were significantly reduced in *P2rx5*-deficient mice.
[62]	2021 Chen et al.	Bone	NLRP3 deficiency reduced the number of osteoclast precursors and bone loss in ligature-induced periodontitis.
[64]	2016 Takahashi et al.	Bone	Genetic and chemical deletion of TRPV1 exacerbated ligature-induced bone loss. Neuronal TRPV1 signaling in periodontal tissue is crucial for the regulation of osteoclastogenesis via the neuropeptide CGRP.
[65]	2017 Zhang et al.	Bone	The expression of miR-335-5p decreased significantly in the periodontal tissue of EP. Overexpression of miR-335-5p in vivo weakened the periodontal bone destruction and inflammation compared to WT-EP group.
[69]	2019 Goes et al.	Bone	Osteocyte specific *Dkk1*-deficient mice significantly prevented ligature-induced bone loss and mitigated inflammation. Dkk-1 derived from osteocytes played a crucial role in alveolar bone loss in periodontitis.
[71]	2016 Duan et al.	Bone	Female mice displayed significantly increased periodontal bone loss, accompanied by elevated expression of proinflammatory cytokines and higher numbers of oral bacteria.
[72]	2020 Aung et al.	Bone	Aged mice showed severe bone loss associated with increased osteoclast activity compared to young mice. Aging-induced impairment of MSC function is potentially correlated with progressive periodontal tissue deterioration.
[73]	2018 Wong et al.	Bone	Micro-CT analysis revealed ligature-induced bone loss in peri-implant sites, such as periodontitis. The ligature was removed after one week, and the periodontitis group experienced significant bone gain, whereas the peri-implantitis group did not.
[75]	2020 Rosset et al.	PDL	Inhibition of transglutaminase activity increased total collagen and thick collagen fiber content in the group with ligation at five days.
[80]	2020 Guo et al.	PDL	Autophagy was significantly increased, and H19 expression was also significantly upregulated during inflammation in PDLCs of ligated mice.

**Table 2:** Summary of the effect of ligature-induced periodontitis in disease model mice.

Reference No.	Year Author	Disease Model	Main Findings
[86]	2017 Maekawa et al.	Streptozotocin-induced diabetic model	Significantly severe alveolar bone loss was observed in STZ mice compared to WT mice at 7 days post ligation. An increased number of TRAP-positive multinucleated cells were observed at the ligated sites in STZ mice.
[87]	2019 Yu et al.	High-fat diet-induced obesity model	ABL and periodontal osteoclast numbers were not affected by diet regardless of ligation or sham-ligation. Leukocyte and macrophage numbers and protein level of TNF-α in the periodontium and serum IL-6 level were downregulated in periodontitis mice fed a high-fat diet.
[88]	2016 Huang et al.	High-fat diet-induced obesity model	ABL was significantly increased with periodontitis and obesity. F4/80 and MCP1 expression was significantly upregulated in gingival tissues with periodontitis but significantly downregulated in the context of obesity.
[89]	2017 Yu et al.	High-fat diet-induced obesity model	In diet-induced obesity mice, periodontitis increased TG levels. After adjusting confounding effects of postoperative weight loss, periodontitis increased levels of not only TG, but also fasting insulin, HOMA-IR, and HDL in the diet-induced obesity mice.
[90]	2006 Gyurko et al.	Akita mice	Ligature-induced periodontal bone loss was significantly greater in Akita mice compared with WT. Chronic hyperglycemia predisposed to an exaggerated inflammatory response and primed leukocytes for marginalization and superoxide production.
[91]	2016 Liu et al.	*db/db* mice	Pancreatic β-cell failure, with insulin resistance, was observed in *db/db* mice, while periodontitis could aggravate β-cell dysfunction-related features.
[92]	2021 Chen et al.	High-fat diet-induced obesity model	Compared with healthy conditions, periodontitis and a high-fat diet had distinct effects on the gingival metabolome. The metabolomic impact of periodontitis was generally greater in high-fat diet mice than in lean controls.
[93]	2021 Sato et al.	High-fat diet-induced obesity model	Gut dysbiosis-associated metabolites from high-fat diet-fed mice worsen alveolar bone destruction. Obesity increases the risk of periodontal disease by increasing the production of uric acid mediated by gut dysbiosis.
[94]	2020 Kantarci et al.	5xFAD mice	Periodontitis increased neuroinflammation in WT mice and disrupted the neuroinflammatory response in 5xFAD mice, suggesting that microglia are central to the association between periodontal disease and Alzheimer’s disease.
[95]	2021 Qian et al.	Amyloid-β protein precursor (AβPP)/presenilin (PS1) transgenic mice	Ligature-induced periodontitis with *P. gingivalis* LPS injection into the periodontal tissue caused cognitive impairment and a significant reduction in the number of neurons.
[98]	2020 Suh et al.	ApoE knockout mice with a high-fat diet	Mice received ligation with/without *P. gingivalis* LPS showed severe periodontitis, systemic inflammation, and aortic plaque formation. The magnitude of systemic inflammation and aortic plaque formation was notably greater in the ligated mice injected with *P. gingivalis* LPS.
[99]	2020 O’Boyle et al.	C57BL/6 mice with focal cerebral ischemia induced by transient occlusion of the middle cerebral artery	Ligature-induced periodontitis with intravenous injection of LPS from *P. gingivalis* did not affect acute stroke pathology in terms of severity, determined primarily by infarct volume, despite the observation of elevated systemic inflammation.
[100]	2018 Kim et al.	BRONJ and DRONJ models under the administration of zoledronic acid or anti- RANKL Ab	Pre-existing pathologic inflammatory condition exacerbated ONJ development after tooth extraction.
[101]	2018 Handa et al.	Fbn-1C1039G/+ mice (C1039G/+) with Marfan syndrome	Fbn-1c1039G/+ mice exhibited slower wound healing compared to WT mice, but periodontal tissue destruction did not differ between these mice.
[102]	2017 Coyac et al.	Phex gene-null mutant phenotype mice	Bone and cementum mineralization appeared to be similarly disturbed, where the hypomineralized pericellular matrix surrounded cells, and the protein osteopontin accumulated in a tissue-specific manner, most notably in the perilacunar matrix surrounding osteocytes.
[103]	2017 Candeo et al.	BALB/c male mice with Asthma induced by ovalbumin injection	Ligature-induced periodontitis reduced the total number of cells in the bronchoalveolar lavage in a mouse model of asthma.
[104]	2020 Rosa et al.	C57BL/6J mice with chronic obstructive pulmonary disease induced by cigarette extract	Ligature induced periodontitis decreased macrophages, TNF-α, and INF-γ expression in bronchoalveolar lavage.
[108]	2019 Hays et al.	Pregnant C57BL/6 mice	The pregnant mice with ligation and *P. gingivalis* administration developed more severe alveolar bone loss.
[109]	2016 Anbinder et al.	BALB/c with ovariectomy	Combination of periodontitis and ovariectomy induced a significantly higher femoral and mandibular bone loss than periodontitis or ovariectomy alone.
[110]	2021 Arjunan et al.	C57BL/6J mice with Laser-induced choroidal neovascularization	An increase in mRNA expression related to oxidative stress, angiogenesis, and pro-inflammatory mediators in the retinae was observed, whereas antioxidant and anti-inflammatory-related gene expression was notably decreased.
[111]	2020 Bi et al.	C57BL/6 male mice with unilateral ureteral ligation	Periodontitis increased mRNA expression of TNFα and IL-1β in the kidneys. Fibrotic areas in the kidneys in the mice with periodontitis were slightly, but not significantly, greater than those without periodontitis.

**Table 3:** Summary of systemic effects of periodontitis found using ligature model.

Reference No.	Year Author	Type of System	Main Findings
[113]	2020 Ribeiro et al.	Cardiovascular system	Heart rate and arterial pressure variability were higher in mice with periodontitis. The mice with periodontitis showed decreased cardiac output and ejection fraction associated with increased myocardial cytokines.
[114]	2020 Zhan et al.	Blood system	Experimental periodontitis induced platelet activation and platelet–leucocyte interaction.
[115]	2021 Han et al.	Blood system	Lower red blood cell counts, hemoglobin, and hematocrit were detected in ligature-induced mice, whereas the levels of hepcidin mRNA expression and serum hepcidin concentrations increased.
[116]	2016 Matsuda et al.	Digestive system	Ligation did not change the insulin sensitivity; it only affected the expression of *Pck1* and *Acaca* in the liver. Ligature placement weakly affected the composition of gut microbiota and gene expression in the intestines.
[117]	2021 Wang et al.	Central nervous system	Microgliosis and astrogliosis were notable in the ligated mice, and the number of glial cells displayed a positive correlation between the degree of periodontal inflammation. TNF-α was highly expressed in the hippocampus of ligated mice.
[118]	2020 Furutama et al.	Central nervous system	In the hippocampus, the *IL-1β* expression levels were significantly increased by ligature-induced periodontitis. The ligation decreased the *Cldn5* expression levels in the hippocampus, and the neutralization of IL-6 restored its levels. The blood-brain barrier was disrupted by ligature treatment.
[119]	2020 Xue et al.	Central nervous system Digestive system	Long-term ligature placement caused progressive cognitive deficits, cerebral neuronal injury, synaptic injury, and glial activation. Ligation disrupted the intestinal barrier and blood-brain barrier and increased the brain LPS levels, TLR4 expression, NF-κB nuclear translocation, and proinflammatory cytokine mRNA levels. Ligated mice exhibited significant dysbiosis of the oral and gut microbiota.
[120]	2020 Kitamoto et al.	Digestive system	Ligation in mice leads to expansion of *Klebsiella* and *Enterobacter* species in the oral cavity and translocation to the gut. The inflammation was triggered by the immigration of oral pathobionts and enhanced by the immigration of oral pathobiont-reactive Th17 cells in parallel.

**Table 4:** Summary of innate immunity in ligature-induced periodontitis model in mice.

Reference No.	Year Author	Cells and Receptors and Complements	Main Findings
[129]	2020 Chadwick et al.	Neutrophils	Neutrophils in healthy oral cavity had the highest expression of CD11a and CD66a. The expression of CD11b was further upregulated by experimental periodontitis.
[11]	2021 Fine et al.	Polymorphonuclear neutrophils (PMNs)	In periodontitis model in mice, PMNs were found to be elevated in the gingiva and bone marrow. The number of PMNs in the blood and colon of mice with induced periodontitis and peritonitis increased more than that in mice with acute peritonitis alone.
[131]	2020 Kim et al.	Neutrophils	Neutrophils in gingival tissue of periodontitis induced RNAKL expression, leading to the early formation of osteoclasts.
[133]	2016 Sima et al.	Polymorphonuclear neutrophils (PMNs)	In the *Nrf2*^−/−^ mouse model of ligation-induced periodontitis, an increase in 8-OHdG–positive cells and more severe alveolar bone loss and breakdown of periodontal tissue were found.
[134]	2021 Pathak et al.	Macrophages	Ligation-induced periodontitis in anti-Act1 mice exhibited a higher level of infiltration of macrophages in periodontal tissue and polarization of M1 macrophages. A higher degree of bone and tissue destruction with an increase in osteoclasts were also found.
[135]	2019 Zhuang et al.	Macrophages	After treatment with CCL2, the loss of alveolar bone and the formation of osteoclasts in the ligation area were significantly reduced. M1/M2 ratio was also reduced.
[136]	2016 Yamaguchi et al.	Macrophages	Transfer of M1 significantly inhibited bone loss and induction of TRAP-positive cells in mice with ligature-induced periodontitis. However, the transfer of M0 or M2 macrophages failed in achieving this efficacy.
[138]	2017 Qin et al.	Lymphoid cells	All subtypes of innate lymphocytes were present in gingival tissues that were healthy or affected by periodontal disease. In periodontitis, the expression of IL-33, as well as ILC2s, were significantly increased through the regulation of AMPK.
[139]	2019 Zheng et al.	Solitary chemosensory cells (SCC)	Induction of periodontitis by ligation in mice lacking SCC function showed more severe alveolar bone loss and gingival inflammatory infiltration. Meanwhile, colonization of the ligation sites was characterized by high bacterial load, low diversity, and high pathogen levels. Local activation of SCC function enhanced expression of β-defensin-3 (Defb3) in gingiva of mice to reduce bacterial load.
[141]	2014 Lin et al.	TLR (TLR2 & TLR)	Alveolar bone loss produced by ligation-induced periodontitis is independent of TLR2 or TLR4. Elevated IL-1β and TNF-α with reduced IL-10 were also observed in all ligated models in mice (WT, *Tlr2* KO, *Tlr4* KO).
[126]	2017 Lin et al.	TLR (TLR2 & TLR)	RNAKL protein expression and significant alveolar bone loss were observed in four mouse models of ligature-induced periodontitis (WT, *Tlr2* KO, *Tlr4* KO, *Tlr2*, and *Tlr4* KO).
[142]	2017 Crump et al.	TLR (TLR9)	Loss of alveolar bone and gingival inflammation were suppressed in *Tlr9*^−/−^ mice, while the expression of A20 mRNA in the gingiva was increased.
[144]	2019 Li et al.	A20	Partial absence of A20 led to more severe alveolar bone loss and inflammatory cell infiltration in a ligature-induced periodontitis mouse model.
[145]	2013 Gao et al.	ChemR23 (chemokine-like receptor 1)	In chemR23tg mice, ligation-induced periodontitis-related alveolar bone resorption was reduced.
[146]	2012 Abe et al.	Complement C5a receptor (C5aR; CD88)	Mice with periodontitis treated with C5aRA at the ligation site showed approximately 50% reduction in bone loss and reduced levels of pro-inflammatory factors compared to the PBS-treated group.
[147]	2014 Maekawa et al.	The third component of complement (C3)	In C3-deficient mice, the loss of alveolar bone caused by periodontitis was suppressed.

**Table 5:** Summary of acquired immunity in ligature-induced periodontitis model in mice.

Reference No.	Year Author	Cell and ILs	Main Findings
[151]	2018 Tsukasaki et al.	Th17 cells	Pathogenic Th17 cells prevented periodontitis progression by inducing mucosal immune response, alveolar bone damage, and tooth loss.
[152]	2021 Pacheco et al.	IL-17	In periodontitis model in mice, *Il17a* expression correlates with the time point of ligation induction. IL-17A antibody reduced IL-6 expression, osteoclastic activity, and alveolar bone loss in murine models.
[153]	2020 Sun et al.	IL-17	Higher levels of *Il17* and IL-17-associated chemokines and cytokines were observed in IL-10-deficient mice with periodontitis. Moreover, the polarization of M1 macrophages and osteoclast differentiation activity in the gingiva were increased.
[154]	2013 Gonçalves-Zillo et al.	IL-17	On day 21, increased IL-17 secretion was observed in all periodontitis mouse models. However, no significant changes in IL-10 and TNF- α levels were observed in *Bdkrb1*^−/−^ mice.
[155]	2015 Abe et al.	B cells	Alveolar bone loss was significantly ameliorated in B-cell-deficient mice. After using APRIL and BLyS antibodies, significant inhibition of B cells in gingival tissue and alveolar bone resorption was observed.
[156]	2017 Yu et al.	B10 cells	Local injection of CpG with CD40L into the gingiva induced B10 cell activity, which increased IL-10 mRNA expression and significantly inhibited alveolar bone loss and expression of pro-inflammatory factors in the gingiva.
[157]	2018 Zhao et al.	B10 cells	In ligation-induced periodontitis in *Tlr9*-deficient mice, treatment by CpG ODNs in combination with CD40L still significantly improved periodontal destruction.
[158]	2017 Hu et al.	B10 cells	Using co-stimulatory molecules (CD40L, IL-21, and anti-Tim-1 mAb) for B10 cells in ligation-induced periodontitis mice could also upregulate the mRNA expression of gingival IL-10 while decreasing the expression of RANKL.

**Table 6:** Summary of cytokines, molecules, and genes in immune responses in ligature-induced periodontitis model in mice.

Reference No.	Year Author	Cytokines and Molecules and Genes	Main Findings
[159]	2019 Bi et al.	β6 integrin	The absence of integrin αvβ6 in the junctional epithelium could lead to the downregulation of the *Aim2* inflammasome and anti-inflammatory IL-10.
[160]	2017 Hirschfeld et al.	Macrophage migration inhibitory factor (MIF)	In *Mif*-deficient mice, the compensatory upregulation of IL-6 and the downregulation of corticosterone levels were observed. However, the release of MMP2 in the periodontal tissue of mice did not seem to be affected by MIF.
[161]	2017 Papadakou et al.	Vascular endothelial growth factor C (VEGFC)	In K14-VEGFC mice, lymphatic vessel hyperplasia occurred, but the severity of bone loss and inflammation did not decrease in the experimental periodontitis.
[12]	2010 Ohnishi et al.	Cot/Tp12	Compared to WT mice, less alveolar bone loss, osteoclast formation, and expression of TNF-α were observed in ligation-induced periodontitis in *cot/tp12*-deficient (*cot/tp12*^−/−^) mice.
[162]	2018 Zhou et al.	MicroRNA-21	In the MicroRNA21 knockout mice with periodontitis, alveolar bone resorption and inflammatory infiltration in gingival were both elevated.
[163]	2018 Ouhara et al.	Human antigen-R (HuR)	HuR stabilizes IL-6 in gingival tissue. Quercetin, as a HuR inhibitor, can lower the level of bone resorption in ligature-induced periodontitis mice model.
[167]	2016 Suzuki et al.	Glucose-dependent insulinotropic polypeptide (GIP)	*Gipr*-knockout mice with ligature-induced periodontitis also showed a significant increase in the gene expression of gingival inflammatory cytokines, TNF-α, and inducible nitric oxide synthase (iNOS), as compared to WT mice with experimental periodontitis.
[168]	2013 Rajshankar et al.	Protein tyrosine phosphatase-α (PTPα)	Loss of alveolar bone, reduction in gingival lamina propria thickness, and collagen fibril number were suppressed in *PTPα*^−/−^ mice.

**Table 7:** Summary of oral and gut microbiota of ligature-induced periodontitis model.

Reference No.	Year Author	Target	Method	Main Findings
[169]	2020 Kittaka et al.	Oral microbiota	16S rDNA amplicon sequencing	The most predominant bacteria in ligatures in a mouse model of cherubism were Pasteurellales. The most predominant bacteria could be a trigger for the initiation of jawbone destruction in human cherubism.
[170]	2020 Williams et al.	Oral microbiota Gut microbiota	16S rDNA amplicon sequencing	The normal oral microbiota in mice protects against inflammation-induced osteonecrosis.
[119]	2020 Xue et al.	Oral microbiota Gut microbiota	16S rDNA amplicon sequencing	Proteobacteria were the most abundant phylum detected in both ligature-induced periodontitis mice and mice without periodontitis, although Proteobacteria were significantly less abundant in ligature-induced periodontitis mice compared with mice without periodontitis.
[171]	2020 Hiyoshi et al.	Oral microbiota	Counts of colony-forming units	Hinokitiol treatment significantly inhibited the alveolar bone loss and osteoclast differentiation induced by tooth ligation.
[172]	2020 Kim et al.	Oral microbiota	Real-time PCR	An oral care probiotic, Weissella cibaria CMU, reduced periodontal tissue destruction by regulating the inflammatory cytokines and by reducing oral bacteria in ligature-induced periodontitis mice.
[173]	2020 Huang et al.	Gut microbiota	16S rDNA amplicon sequencing	Periodontitis led to gut microbiota dysbiosis in mice with hyperlipidemia. Non-surgical periodontal treatment normalized the gut microbiota.
[174]	2021 Li et al.	Gut microbiota	16S rDNA amplicon sequencing	Parabacteroides and Desulfovibrionaceae increased, and several butyrate-producing bacteria decreased significantly in the gut microbiota of ligature-induced periodontitis mice compared to control mice.

**Table 8:** Summary of ligature-induced periodontitis models with bacterial factors.

Reference No.	Year Author	Periodontal Enhance Factor	Main Findings
[9]	2021 Akkaoui et al.	Ligation with injection of LPS from *P. gingivalis*	*P. gingivalis*-LPS mice elevated the pro-inflammatory cytokines compared to control mice.
[175]	2021 An et al.	Ligation with oral feeding of *P. gingivalis*	A monoclonal antibody that targeted the DHYAVMISK peptide might protect against a *P. gingivalis* infection.
[176]	2017 Bhattarai et al.	Ligation with injection of LPS from *P. gingivalis*	Genistein protected against alveolar bone loss and periodontal tissue destruction in LPS/ligature-induced periodontitis mice.
[177]	2021 Cao et al.	A ligature inoculated with *P. gingivalis*	Local transfer of CD19^+^ CD1d^hi^ CD5^+^ B cells could inhibit attenuate alveolar bone loss in periodontitis mice by ligatures inoculated with *P. gingivalis*.
[178]	2021 Clark et al.	A ligature inoculated with *P. gingivalis*	The age-related changes to the macrophage contributed to the pathogenesis of periodontal disease.
[179]	2018 Francis et al.	A ligature inoculated with *P. gingivalis*	Keratinocyte-specific ablation of protease-activated receptor 2 prevented the increase in the number of osteoclasts and the up-regulation of the inflammatory markers in ligature-induced periodontitis mice.
[180]	2019 Ideguchi et al.	A ligature inoculated with *P. gingivalis*	Levels of serum IgG antibody against *P. gingivalis* were significantly higher in the ligature + *P. gingivalis* group than in the *P. gingivalis* + glycyrrhizic group.
[181]	2016 Lapérine et al.	A ligature inoculated with *P. gingivalis*	IL-33 expression of periodontitis induced by a *P. gingivalis*-soaked ligature was increased in gingival epithelial cells similarly as in human CP.
[182]	2017 Nagashima et al.	A ligature inoculated with *P. gingivalis*	Periodontal CXC-chemokine receptor 4 signaling in several cell types such as fibroblasts, macrophages, osteoblasts, and osteoclasts in *P. gingivalis*-induced periodontitis depresses alveolar bone resorption in periodontitis.
[183]	2020 Wang et al.	A ligature inoculated with *P. gingivalis*	NIK-SMI1 treatment resulted in attenuated periodontitis progression and pro-inflammatory cytokines expression in periodontitis mouse model.
[184]	2020 Wang et al.	A ligature inoculated with *P. gingivalis*	Halofuginone significantly reduced the expression levels of pro-inflammatory cytokines, and markedly suppressed immune cell infiltration into the infected sites.
[185]	2021 Wang et al.	A ligature inoculated with *P. gingivalis*	The local infiltration of B10 cells into periodontal tissue promoted anti-inflammatory responses in ligature-induced periodontitis mice.
[186]	2017 Wang et al.	A ligature inoculated with *P. gingivalis*	The adoptive transfer of B10 cells alleviated periodontal inflammation and bone loss in experimental periodontitis mice.
[187]	2020 Korah et al.	A ligature inoculated with *P. gingivalis*	Osteonecrosis area and osteoclast number were significantly elevated in Msx2 knock-in periodontitis mice compared with wild-type periodontitis mice.
[188]	2007 Li et al.	A ligature inoculated with *P. gingivalis*	The ligature inoculated with *P. gingivalis* group showed significantly increased epithelial downgrowth, inflammation, alveolar bone loss, and osteoclast activity throughout the experimental period compared to the controls.
[189]	2019 Palioto et al.	Ligation with *P. gingivalis* gavage	Ligature, *P. gingivalis* gavage, and ligature with *P. gingivalis* gavage groups induced significant bone loss compared to the sham group. In terms of the inflammatory markers and epigenetic changes, the alteration of *P. gingivalis* gavage and ligature with *P. gingivalis* gavage groups were significantly higher than that of local ligature-induced periodontitis.
[10]	2019 Qi et al.	A ligature inoculated with *P. gingivalis*	Inhibition TLR4 in vivo significantly improved the alveolar bone resorption in periodontitis model mice.
[190]	2021 Wang et al.	A ligature inoculated with *P. gingivalis*	Treatment of TPCA-1 inhibited the osteoclastogenesis through the inactivation of NF-κB pathway in mouse chronic periodontitis model.
[191]	2016 Yang et al.	A ligature inoculated with *P. gingivalis*	Periodontitis mice induced by ligation with *P. gingivalis* showed significantly more severe alveolar bone loss than control mice.
[192]	2019 Sulijaya et al.	Ligation with oral feeding of *P. gingivalis*	KetoC attenuated alveolar bone destruction and suppressed the abundance level of *P. gingivalis* in the ligature-induced periodontitis mice.
[193]	2018 Duan et al.	Ligation combined with *P. gingivalis* infection	Ligation itself did not cause higher gingival inflammation and bone loss in pregnant mice than non-pregnant mice, while ligation combined with *P. gingivalis* infection led to increased gingival inflammation and periodontal bone loss.
[194]	2000 Kimura et al.	A ligature inoculated with *P. gingivalis*	The ligature + *P. gingivalis* mice showed alveolar bone loss in the infected sites which was found to be greater than that in the periodontitis mice induced by ligation alone.
[195]	2018 Bugueno et al.	A ligature inoculated with *P. gingivalis*	An increased expression of apoptotic peptidase activating factor 1 was observed in a murine experimental periodontitis model induced by *P. gingivalis* soaked ligatures.
[200]	2016 Kubota et al.	Ligation with injection of nicotine and cigarette smoke condensate	Nicotine and cigarette smoke condensate promoted periodontal destruction in mice with a ligature, accompanied by increased osteoclastogenesis and RANKL expression.

**Table 9:** Summary of the effect of new treatment methods in ligature-induced periodontitis.

Reference No.	Year Author	Treatment	Main Findings
[203]	2017 Shi et al.	Systemic antibiotics	Systemic antibiotic administration reduced bone loss. Gingival mRNA and protein RANKL/OPG expression ratio was decreased.
[204]	2020 Maekawa et al.	Erythromycin	ERM suppressed inflammation and inhibited bone loss via activation of DEL-1 expression.
[205]	2021 Tamura et al.	Erythromycin	ERM inhibited alveolar bone loss by increasing Del1 expression and decreasing the expression of osteoclast-related factors.
[211]	2017 Wisitrasameewong et al.	Anti-DC-STAMP-mAb	Local injection and systemic administration of anti-DC-STAMP-mAb significantly downregulated the alveolar bone loss. DC-STAMP promotes local osteoclast cell fusion without affecting adaptive immune responses to oral bacteria.
[214]	2017 Maekawa et al.	Secreted frizzled-related protein 5 (sFRP5)	Local sFRP5 administration inhibited inflammation and bone absorption and decreased the number of osteoclasts in bone tissue.
[217]	2019 Tamura et al.	Rice endosperm protein (REP) 9 and 11	Local treatment with REP 9 and 11 inhibited the activity of inflammatory and osteoclast-related molecules and significantly decreased bone resorption.
[218]	2019 Aoki-Nonaka et al.	Amyl-1-18	Amyl-1-18 prevented alveolar bone destruction via suppression of LPS-induced inflammatory cytokine production.
[219]	2015 Mizuno et al.	Reveromycin A (RMA)	RMA treatment decreased osteoclasts, alveolar bone loss, attachment loss, and inflammatory cytokine expression.
[226]	2016 Huang et al.	Myricetin	Myricetin inhibited osteoclast formation and prevented alveolar bone loss in an OVX mouse model.
[230]	2019 Adhikari et al.	Oleanolic acid acetate (OAA)	OAA induced bone formation and remodeling via proper modulation of osteoblasts, osteoclasts, and inflammation by regulating TGF-β and Wnt signaling.
[233]	2020 Kim et al.	6-Shogaol	Administration of 6-shogaol prevented osteoclastogenesis and alveolar bone resorption, decreased the number of macrophages and neutrophils, and downregulated the expression of IL-1β and TNF-α in periodontal tissue.
[234]	2017 Ihn et al.	A novel pyrimidine compound (OCLI-023)	OCLI-023 inhibited ligature-induced bone loss and reduced the number of osteoclasts induced by periodontitis.
[235]	2018 Ihn et al.	A novel benzamide-linked derivative (OCLI-070)	OCLI-070 inhibited osteoclast formation and differentiation, reduced *Nfatc1* and the expression of osteoclast-specific genes, and prevented bone resorption via the suppression of RANKL-mediated ERK and NF-κB signaling pathways.
[238]	2020 Jiang et al.	LLP2A-alendronate (LLP2A-Ale)	LLP2A-Ale stimulated alveolar bone formation, reversed bone loss, and reduced the levels of periodontitis-induced circulating inflammatory cytokines.
[239]	2020 Cafferata et al.	Boldine	Boldine decreased osteoclast numbers and the RANKL/OPG ratio in periodontal sites. The Th17-lymphocyte detection and response were reduced, and the Treg-lymphocyte detection and response were increased by boldine.
[240]	2015 Zhen et al.	Resveratrol	Blood glucose levels were decreased, alveolar bone loss was ameliorated, and high levels of IL-1β, IL-6, IL-8, TNF-α, and TLR4 were suppressed by resveratrol in the gingival tissue.
[241]	2018 Ikeda et al.	Resveratrol derivative-rich merinjo (MSE)	MSE decreased oxidative stress, inhibited M-CSF/sRANKL-mediated osteoclast formation, and downregulated osteoclast activity on the periodontal side.
[47]	2019 Akutagawa et al.	Glycyrrhizic acid	Glycyrrhizic acid suppressed the release of inflammatory cytokines, high mobility group box 1, and receptor for AGE, which were increased by ligature/*P. gulae* at the mRNA level in gingival tissue and the protein level in the serum of diabetic mice.
[242]	2020 Kaboosaya et al.	Green tea	Systemic administration of green tea relieved alveolar bone loss and reduced the number of inflammatory cells and osteoclasts.
[243]	2016 Cui et al.	Scaling and root planing	Prevention of alveolar bone loss, improvement of lipid profile, and inhibition of systemic inflammation with reduced plasma IL-6 levels were observed.
[244]	2020 Ying et al.	Low-intensity pulsed ultrasound (LIPUS)	LIPUS treatment alleviated alveolar bone homeostasis in periodontitis by downregulating oxidative stress via PI3K-Akt/nuclear factor erythroid 2-related factor (NRF2) signaling.
[245]	2021 Snipes et al.	Inhibitor of sphingosine-1-phosphate receptor 2 (JTE013)	JTE013 attenuated ligature-induced alveolar bone loss and reduced inflammation-related gene expression levels.
[246]	2019 Kure et al.	IkB kinase inhibitor (IMD-0354)	IMD-0354 suppressed bone loss and RANKL gene expression levels in gingival tissue by downregulating NF-κB.
[247]	2021 Ihn et al.	Fatty acid amide hydrolase inhibitor (PF-3845)	PF-3845 demonstrated anti-osteoclastogenic and anti-resorptive activities by suppressing the phosphorylation of rapidly accelerated fibrosarcoma (RAF), mitogen-activated protein kinase (MEK), extracellular signal-regulated kinase (ERK), and NF-κB inhibitor (IκBα).
[248]	2021 Tanaka et al.	DNA methyltransferase inhibitor (Decitabine)	Decitabine inhibited osteoclastogenesis through the upregulation of anti-inflammatory cytokines via Krüppel-like factor-2 (KLF2)-dependent mechanisms.
[249]	2019 Oliveira et al.	Renin inhibitor (Aliskiren)	Aliskiren treatment reduced gene expression of renin-angiotensin system (RAS) components in the gingival tissue of normal and diabetic mice. Administration of aliskiren also downregulated the gene expression of COL1A1, COL1A2, and fibronectin that was increased by periodontitis.
[98]	2020 Suh et al.	Treatment of atherosclerosis (Rosuvastatin)	Rosuvastatin inhibited the TNF-α-induced osteoclast formation, endothelial cell phenotypic changes, foam cell formation, and the expression of CD47 and other oncogenes in arterial smooth muscle cells related to atherosclerosis.
[250]	2017 Iwamatsu-Kobayashi et al.	Metal ions from S-PRG filler	The S-PRG metal ion suppressed the destruction of the collagen bundle in the periodontal ligament and inhibited the infiltration of inflammatory cells. The number of infiltrating neutrophils and macrophages was significantly suppressed.
[251]	2016 Yu et al.	pH-responsive hydrogel with an anti-glycation agent (PTB)	PTB significantly reduced periodontal bone levels (PBL) and inflammatory cell infiltration in experimental periodontitis plus hydrogel with PTB during the induction phase. PTB significantly decreased PBL and inflammatory cell infiltration, and significantly greater collagen deposition was observed in both hydrogel groups with and without PTB at 4 and 14 days after removing ligature.
[252]	2017 Iguchi et al.	Local bone marrow stromal cell (BMSC)	Alveolar bone loss around the ligated molars was significantly reduced in BMSC-treated mice. Inflammatory infiltration was also decreased, and the number of TRAP-positive osteoclasts was notably increased by BMSC treatment.
[253]	2021 Nakao et al.	Exosomes from TNF-alpha-treated human gingiva-derived MSCs (GMSCs)	Local injection of GMSC-derived exosomes significantly decreased bone loss and the number of TRAP-positive osteoclasts, and these effects were further enhanced by preconditioning of GMSCs with TNF-α. The exosome with TNF-α reduced RANKL mRNA expression and significantly elevated OPG mRNA expression.
[254]	2020 Nagai et al.	Hydrogel-formulated inhibitor of prolyl-4-hydroxylase (1,4-DPCA/hydrogel)	Injection of 1,4-DPCA/hydrogel increased gingival hypoxia-inducible factor 1α (HIF-1α) protein levels and alveolar bone regeneration. The 1,4-DPCA/hydrogel-induced regeneration of alveolar bone was associated with enhanced expression of osteogenic genes, decreased expression of inflammation-related genes, and increased abundance of Tregs in the periodontal tissue.

## Data Availability

Not applicable.
